# Exploring the Bioactive Properties of *Thymus kotschyanus* var. *kotschyanus* and Endemic *Thymus brachychilus*: From Plant Extracts to Alginate Film Formulations for Wound Infection Management

**DOI:** 10.3390/pharmaceutics18101298

**Published:** 2026-10-14

**Authors:** Erkan Rayaman, Fatma Betül Zengin, Duygu Taşkın, Ceyda Ekentok Atıcı, Ömer Kılıç, Elif Çalışkan Salihi, Turgut Taşkın, Zeynep Şahan, Hatice Kübra Elçioğlu

**Affiliations:** 1 Department of Pharmaceutical Microbiology, Faculty of Pharmacy, Marmara University, Istanbul 34854, Türkiye; 2 Marmara Pharmacy Drug and Innovative Product Development Unit, Faculty of Pharmacy, Marmara University, Istanbul 34854, Türkiye; elif.caliskan@marmara.edu.tr (E.Ç.S.); turguttaskin@marmara.edu.tr (T.T.); kubra.elcioglu@marmara.edu.tr (H.K.E.); 3 Department of Pharmacognosy, Faculty of Pharmacy, Marmara University, Istanbul 34854, Türkiye; fatma.zengin@marmara.edu.tr; 4 Department of Analytical Chemistry, Faculty of Pharmacy, University of Health Sciences, Istanbul 34668, Türkiye; duygu.taskin@sbu.edu.tr; 5 Department of Pharmaceutical Biotechnology, Faculty of Pharmacy, Marmara University, Istanbul 34654, Türkiye; ceyda.ekentok@marmara.edu.tr; 6 Department of Pharmaceutical Botany, Faculty of Pharmacy, Adıyaman University, Adıyaman 02040, Türkiye; okilic@adiyaman.edu.tr; 7 Department of Basic Pharmaceutical Sciences, Faculty of Pharmacy, Marmara University, Istanbul 34854, Türkiye; 8 Department of Veterinary, Kahta Vocational College, Adıyaman University, Adıyaman 02040, Türkiye; zsahan@adiyaman.edu.tr; 9 Department of Pharmacology, Faculty of Pharmacy, Marmara University, Istanbul 34854, Türkiye

**Keywords:** *Thymus kotschyanus* var. *kotschyanus*, *Thymus brachychilus*, antimicrobial activity, anti-quorum sensing activity, antibiofilm activity, anti-infective activity, alginate, wound healing

## Abstract

**Background/Objectives:** Antibiotic resistance poses significant challenges to the treatment of infectious diseases. Wounds occur as a result of damage to the skin caused by various factors and may become life-threatening with the development of infection. Plants can exhibit considerable microbiological activity due to their rich phytochemical composition. *Thymus kotschyanus* var. *kotschyanus* and the endemic species *Thymus brachychilus* collected from Adıyaman Province, Türkiye, have been widely used by local communities for their therapeutic properties and other purposes. **Methods**: In this study, ethanol extracts of these plants were prepared and evaluated for their antimicrobial activity against nine Gram-negative bacteria, seven Gram-positive bacteria, one mycobacterial strain, and four yeast strains using agar well diffusion and microdilution methods. Their antibiofilm activity was assessed using a microplate-based assay, anti-quorum sensing activity was evaluated using *Chromobacterium violaceum* strains, anti-infective activity was investigated using the *Caenorhabditis elegans* model, and cytotoxicity was evaluated using the MTT assay. The phytochemical composition of the extracts was determined by HPLC-DAD. Alginate film formulations containing the extracts were prepared using the solvent-casting method and characterized by FTIR spectroscopy and light microscopy. Their antimicrobial and antibiofilm activities were subsequently evaluated. **Results**: Both extracts exhibited antimicrobial, antibiofilm, anti-quorum sensing, and anti-infective activities, while no cytotoxic effects were observed. Rosmarinic acid was identified as the major compound in both extracts. The extract-loaded alginate films exhibited antimicrobial and antibiofilm activities and showed no cytotoxic effects. **Conclusions**: These findings suggest that alginate film formulations may be a promising approach for the topical application of *T. kotschyanus* var. *kotschyanus* and *T. brachychilus* extracts. In particular, the alginate films containing both plant extracts may have potential as wound dressing materials for preventing wound infections or supporting the treatment of infected wounds.

## 1. Introduction

Infectious diseases have remained a major global health challenge throughout human history. The rapid spread of antimicrobial resistance has further exacerbated this problem, leading to the emergence of infections that are increasingly difficult or even impossible to treat with currently available antimicrobial agents. As a result, the number of such infections continues to rise worldwide each year [1,2,3].

The development of infectious diseases depends on several factors related to microbial pathogenicity. After adhering to a surface, microorganisms adapt to their environment and begin to proliferate when conditions are favorable. Some microorganisms, upon reaching a critical population density, communicate through the quorum sensing system [4] and produce various products known as virulence factors. One of the most important of these factors is biofilm. Biofilm is an extracellular polymeric matrix composed of extracellular DNA, polysaccharides, proteins, lipids, and water that surrounds microbial cells. This structure protects microorganisms from a wide range of external environmental stresses. Moreover, antimicrobial agents administered for the treatment of infectious diseases often fail to penetrate the biofilm matrix at effective concentrations. Consequently, biofilm formation contributes to the development of antimicrobial resistance and chronic infections [4,5,6,7,8].

In addition to the identification of novel antimicrobial compounds for the development of alternative strategies against infectious diseases, the discovery of substances capable of inhibiting microbial adhesion, suppressing or disrupting quorum sensing (QS) systems, preventing biofilm formation, and exhibiting anti-infective activity is of considerable importance [9,10]. Owing to their rich chemical diversity, plants represent a valuable source of bioactive compounds with the potential to exert these effects and are therefore extensively investigated for such purposes [1,11].

*Caenorhabditis elegans* is a free-living soil nematode measuring approximately 1–2 mm in length. Due to its susceptibility to a wide range of human pathogens, *C. elegans* has been widely employed as a model organism in studies evaluating anti-infective and antimicrobial activities [12,13,14,15,16].

The skin is the largest organ of the human body. Despite its robust structure and various protective mechanisms, it remains vulnerable to infections due to its constant exposure to the external environment, the complexity of its resident microbiota, and its frequent contact with pathogenic microorganisms. Furthermore, skin susceptibility to infections may increase as a result of impaired skin integrity, aging, cancer, or immunosuppression associated with chronic diseases, particularly diabetes mellitus. Diabetic patients and elderly individuals with chronic diseases constitute the major risk groups for skin infections. If left untreated or inadequately treated, skin infections may spread to deeper tissues, allowing the causative microorganisms to enter the bloodstream and potentially result in sepsis or dissemination of the infection to other parts of the body, thereby posing a serious threat to life. Although skin infections can be caused by viruses, bacteria, and fungi, the most frequently encountered pathogenic microorganisms include *Staphylococcus* spp., *Pseudomonas* spp., *Corynebacterium* spp., *Enterococcus* spp., *Streptococcus* spp., *Cutibacterium* spp., *Acinetobacter baumannii*, *Escherichia coli*, various filamentous fungi, and yeasts such as *Candida albicans*. Some of these microorganisms are also members of the normal skin microbiota but may contribute to infection development through the expression of virulence factors, particularly adhesion, quorum sensing, and biofilm formation [17,18,19,20,21,22]. Therefore, in addition to possessing antimicrobial activity, the identification of compounds capable of inhibiting microbial adhesion, disrupting quorum sensing systems, preventing or reducing biofilm formation, and exhibiting anti-infective activity, as well as the development of suitable formulations containing such compounds, is of great importance in the fight against infectious diseases.

To enable the use of active pharmaceutical ingredients or naturally derived products with pharmaceutical activity, they need to be formulated into suitable dosage forms and subjected to appropriate quality controls. Natural film formulations are widely used for substances intended for application to skin conditions such as wounds and acne. Alginate films are among the most commonly used formulation types. Alginate film formulations are frequently preferred for the development of wound care products due to their non-toxic nature, low cost, ease of processing, high biocompatibility, flexibility, protective properties, gas permeability, and, particularly, their ability to preserve antimicrobial activity [23,24,25,26,27,28,29,30]. Plants belonging to the genus *Thymus* L. are members of the *Lamiaceae* family and are widely distributed throughout the world, except in polar regions. They have been extensively used in traditional medicine for centuries and possess considerable commercial value due to their rich content of essential and aromatic oils [31]. The chemical composition of *Thymus* spp. is highly diverse, with major constituents including thymol, carvacrol, linalool, geraniol, borneol, and p-cymene. In addition to their widespread use as herbal teas and culinary spices, members of this genus are employed in medicinal and cosmetic applications. Some studies have demonstrated that *Thymus* taxa exhibit antimicrobial, antioxidant, and enzyme inhibitory activities, highlighting their potential as valuable sources of bioactive compounds [32,33,34,35,36]. These biological properties make *Thymus* species promising candidates for the discovery of novel antimicrobial, antibiofilm, and anti-infective agents.

*Thymus kotschyanus* Boiss et Hohen var. *kotschyanus* is an Irano-Turanian species predominantly distributed in Türkiye and the Caucasus region. In traditional medicine, many *Thymus* members have been widely utilized for the management of respiratory tract infections, including influenza, the common cold, and tonsillitis, as well as for the treatment of gastrointestinal disorders. These ethnopharmacological uses suggest the presence of bioactive constituents with potential therapeutic value [35,36,37,38]. *Thymus brachychilus* Jalas is an endemic species native to Türkiye, for which only limited scientific information is currently available. The species is primarily distributed throughout the Central and Southern Anatolian regions [39]. In addition to its ecological significance, *T. brachychilus* has traditionally been used in the production of certain local cheeses in Türkiye [40,41,42].

The aim of this study was to comprehensively evaluate the antimicrobial, antibiofilm, anti-quorum sensing, anti-infective, and cytotoxic activities of *T. kotschyanus* var. *kotschyanus* and the endemic species *T. brachychilus*. In addition, alginate film formulations containing extracts were developed, and their biological activities were assessed to determine their potential applicability in wound management and infection control.

## 2. Materials and Methods

### 2.1. Plant Material Collection and Identification

*Thymus kotschyanus* var. *kotschyanus* and *Thymus brachychilus* were collected in Adıyaman (Türkiye), from the vicinity of the bridge over the Tilkihan stream (northwest of Şerefli village) towards the Gömükhan dam, inside the canyon, steppe rocky slopes, 800–1000 m, in June 2025 by Dr. Ö. Kılıç and Dr. Z. Sahan. Small amounts were taken from the above-ground part of the plants (not the entire root), without causing any harm to their habitat or future distribution in the natural environment. Then, plant samples were dried according to herbarium techniques and identified by plant taxonomist Dr. Ö. Kılıç, with volume 7 of Flora of Turkey and East Aegean Islands [39]. Plant samples were deposited in the Department of Pharmaceutical Botany, Faculty of Pharmacy, University of Adıyaman, Türkiye (Voucher No: ÖK-ZŞ 3000 and ÖK-ZŞ 3056, respectively).

### 2.2. Preparation of Extracts

The aerial parts of *Thymus kotschyanus* var. *kotschyanus* and *T. brachychilus* were dried at room temperature (25 °C) and subsequently powdered. Twenty grams of each powdered plant sample were weighed and extracted with a total of 1000 mL of ethanol (Merck, Darmstadt, Germany) (250 mL × 4). The maceration process was continued for a total of 96 h. Following maceration, the liquid portions were concentrated under reduced pressure at a low temperature (45 °C) using a rotary evaporator (Heidolph Hei-VAP Expert, Schwabach, Germany) and then lyophilized (Faithful FSF-10N-50C, Ningbo, China) to obtain crude extracts. The extraction yields were calculated as 5.2% for *T. kotschyanus* var. *kotschyanus* (TkE) and 6.3% for *T. brachychilus* (TbE). The extracts were stored at 4 °C until the day the study began. The extracts were prepared in distilled water at a concentration of 15 mg/mL during the experiments and used accordingly.

### 2.3. Antimicrobial Activity

Antimicrobial activity was assessed using the agar well diffusion and broth microdilution methods.

#### 2.3.1. Microorganisms and Cultivation

In our study, microorganisms that cause general and skin infectious diseases were selected. *Pseudomonas aeruginosa* ATCC 27853, *Pseudomonas aeruginosa* PAO1, *Acinetobacter baumannii* ATCC 19606, *Proteus mirabilis* ATCC 14153, *Klebsiella pneumoniae* ATCC 4352, *Salmonella Typhimurium* ATCC 14028, *Escherichia coli* ATCC 25922, *Chromobacterium violaceum* ATCC 12472, *Chromobacterium violaceum* CV026, *Staphylococcus aureus* ATCC 43300, *Staphylococcus aureus* ATCC 29213, *Staphylococcus epidermidis* ATCC 12228, *Staphylococcus epidermidis* ATCC 35984, *Enterococcus faecalis* ATCC 29212, *Streptococcus mutans* ATCC 25175, *Cutibacterium acnes* ATCC 11827, *Mycobacterium smegmatis* ATCC 19420 were the bacterial strains and *Candida albicans* ATCC 90028, *Candida albicans* SC5314, *Candida tropicalis* KUEN 1021, *Candida glabrata* ATCC 90030 were the fungal strains. Bacterial strains were cultured on tryptic soy agar (TSA) (Merck, Darmstadt, Germany). *C. acnes* was incubated under anaerobic conditions at 37 °C for 72 h, *M. smegmatis* at 37 °C for 72 h, *C. violaceum* at 28 °C for 24 h, and all other bacterial strains at 37 °C for 24 h. *Candida* spp. were cultured on Sabouraud dextrose agar (SDA) (Merck, Darmstadt, Germany) and incubated at 37 °C for 48 h.

#### 2.3.2. Agar Well Diffusion Method

The agar well diffusion assay was performed based on European Committee on Antimicrobial Susceptibility Testing (EUCAST) recommendations. Following cultivation (Memmert INE 500, Büchenbach, Germany), microbial suspensions were prepared in sterile physiological saline (SPS) and adjusted to a turbidity equivalent to 0.5 McFarland for bacteria and 1 McFarland for yeasts. The prepared suspensions were spread onto the surface of Mueller–Hinton Agar (MHA) (Merck, Darmstadt, Germany) for bacterial strains and SDA for yeast strains. Wells with a diameter of 5 mm were then punched into the agar media. Subsequently, 50 µL of each sample was added to the wells. The plates were incubated at 37 °C for 24 h for bacteria and 48 h for yeasts. After incubation, the growth inhibition zones formed around the wells were measured in millimeters (mm) using a digital caliper. All experiments were performed in triplicate. SPS, meropenem (10 µg/well), and amphotericin B (100 µg/well) were used as controls [43,44].

#### 2.3.3. Determination of Minimum Inhibitory Concentrations, Minimum Bactericidal Concentrations, and Minimum Fungicidal Concentrations

The microdilution assay was performed based on EUCAST recommendations. Following cultivation as described in the “Microorganisms and Cultivation” section, microbial suspensions were prepared in sterile physiological saline (SPS) and adjusted to a turbidity equivalent to the 0.5 McFarland standard. The suspensions were then diluted to obtain final inoculum concentrations of 5 × 10^5^ CFU/mL for bacteria and 1 × 10^5^–5 × 10^5^ CFU/mL for yeasts. Mueller–Hinton Broth (MHB) was used for bacterial assays, whereas RPMI 1640 medium was used for yeast assays. After dispensing the appropriate culture media into sterile 96-well microplates, two-fold serial dilutions of the extracts were prepared. Subsequently, bacterial or yeast suspensions were added to the corresponding wells, and the microplates were incubated at 37 °C. The incubation period was 18–24 h for bacteria and 24 h for yeasts. Following incubation, the lowest extract concentration showing no visible growth for bacteria or causing a ≥ 90% reduction in yeast growth compared with the growth control was recorded as the MIC. Meropenem for bacteria, amphotericin B for yeasts, and medium and growth controls were included in the experiments. All assays were performed in triplicate [45,46]. For the determination of MBC and MFC values, 10 μL aliquots were taken from wells showing no visible growth in the MIC assay and subcultured onto TSA for bacteria and SDA for yeasts. The inoculated plates were incubated at 37 °C for 24 h for bacteria and 24–48 h for yeasts. The lowest extract concentration that resulted in no microbial growth after subculturing was considered the MBC for bacteria and the MFC for yeasts.

### 2.4. Effect of Extracts on Quorum Sensing Activity

The quorum sensing (QS) inhibitory activity of the extracts and alginate film formulations was evaluated by assessing their effects on violacein production in *C. violaceum* ATCC 12472 and on the biosensor strain *C. violaceum* CV026.

*C. violaceum* ATCC 12472 was cultured, and a bacterial suspension equivalent to a 0.5 McFarland standard was prepared in LB broth (Merck, Darmstadt, Germany). Subsequently, 1 mL of the suspension was inoculated into 9 mL of LB broth containing the extracts at half of the minimum inhibitory concentration (MIC/2), as well as into LB broth without the extracts (control). The cultures were incubated at 28 °C for 24 h in a shaking incubator (N-biotek NB-205L, Bucheon, Republic of Korea). Following incubation, the cultures were centrifuged at 8000 rpm (Sigma 1-14, Osterode am Harz, Germany) for 10 min, and the supernatants were discarded. The resulting pellets were resuspended in 1 mL of n-butanol, vortexed for 30 s, and centrifuged again at 8000 rpm for 10 min. The supernatants were transferred (100 µL) into 96-well microplates, and the absorbance was measured at 585 nm using a microplate reader (Biotek Epoch, Winooski, VT, USA). The percentage change in violacein production was calculated relative to the untreated control. All experiments were performed in triplicate [47,48,49,50]. In parallel with this experiment, the effects of the extracts at the concentrations used under the experimental conditions on the growth of *C. violaceum* ATCC 12472 were evaluated by measuring the optical density at 600 nm (OD600) using a microplate reader at 4 h intervals.

Following cultivation, a suspension of *C. violaceum* CV026 equivalent to a 0.5 McFarland standard was prepared in sterile physiological saline. The suspension was then spread onto the surface of LB agar supplemented with 500 nM N-hexanoyl-L-homoserine lactone (C6-HSL) (Sigma-Aldrich, Darmstadt, Germany). Wells (5 mm in diameter) were punched into the agar, and 50 µL of each extract samples were added to the wells. The plates were incubated at 28 °C for 24 h (Memmert INE 500, Büchenbach, Germany). Quorum sensing (QS) inhibition was determined by measuring the diameter (mm) of the opaque, pigmentless zone consisting of viable bacterial cells surrounding each well using a digital caliper [50,51,52]. All experiments were performed in triplicate.

### 2.5. Effect of Extracts and Alginate Film Formulations on Biofilm Formation

To evaluate the effects of the extracts and alginate film formulations on biofilm formation, *P. aeruginosa* ATCC 27853, *P. aeruginosa* PAO1, *A. baumannii* ATCC 19606, *S. aureus* ATCC 43300, *S. aureus* ATCC 29213, and *S. epidermidis* ATCC 35984 were used, and the previously reported method was followed [30]. Briefly, the microorganisms were cultured as described in the “Microorganisms and Cultivation” section. Bacterial strains were cultured in Tryptic Soy Broth supplemented with 1% glucose (TSBG) (Sigma-Aldrich, Darmstadt, Germany), whereas yeast strains were cultured in RPMI 1640 medium (Sigma-Aldrich, Darmstadt, Germany). The cultures were then transferred to the corresponding fresh media and adjusted to a 0.5 McFarland turbidity standard. Subsequently, 180 µL of each adjusted microbial suspension was transferred into the wells of flat-bottom microplates. Then, 20 µL of the extracts and alginate film formulations dissolved in distilled water were added to the wells at concentrations corresponding to the MIC, 1/2 MIC, and 1/4 MIC values for each experimental series. The plates were incubated under appropriate conditions for each microorganism, namely, 24 h for bacteria and 48 h for yeasts, using a Memmert INE 500 incubator (Büchenbach, Germany). Following incubation, the wells were carefully emptied and washed twice with phosphate-buffered saline (PBS; 250 µL). The biofilms were fixed with 200 µL of 99% methanol (Merck, Darmstadt, Germany) for 15 min and subsequently allowed to dry at room temperature. The wells were then stained with 0.1% crystal violet (Sigma-Aldrich, Darmstadt, Germany) for 5 min and washed with water. Subsequently, 200 µL of 95% ethanol was added to each well to solubilize the crystal violet. The optical density (OD) values were measured at 595 nm using a microplate reader (BioTek Epoch, Winooski, VT, USA). All experiments were performed in triplicate. According to the method applied, the optical density values of the negative and positive control wells were measured, and the mean value was taken. For each strain, the OD value of the positive control (wells containing only the microorganism) was considered as 100%, and the percentage changes in the wells containing different concentrations of the samples were calculated relative to this control well [30,53,54,55].

### 2.6. Detection of Anti-İnfective Effect of Extracts in C. elegans Model

The anti-infective activity of the extracts was evaluated according to a previously reported method [30]. Briefly, *C. elegans* AU37 was used as the model organism and infected with *P. aeruginosa* ATCC 27853 or *S. aureus* ATCC 29213. Synchronized nematodes were exposed to the respective pathogens in nematode growth medium containing the extracts at MIC/2 concentration, with *E. coli* OP50 as the food source. Approximately 20–40 nematodes were transferred to each well of 24-well microplates and incubated at 25 °C. Nematode survival was monitored daily using a stereomicroscope (Olympus SZ61, Kyoto, Japan) until complete mortality. Extract-free medium and *E. coli* OP50 were used as controls [30,56,57,58,59].

### 2.7. Cytotoxic Activity of Extracts and Alginate Film Formulations

The cytotoxic effects of the extracts, extract-free alginate film formulation and extract-loaded alginate film formulations were evaluated using the 3-(4,5-dimethylthiazol-2-yl)-2,5-diphenyltetrazolium bromide (MTT) assay with Cell Proliferation Kit I (MTT kit; Roche), according to the manufacturer’s instructions. Briefly, L-929 (ATCC CCl-1) cells were cultured in DMEM supplemented with 10% FBS and seeded into 96-well plates at a density of 5 × 10^3^ cells/well. After overnight incubation at 37 °C in 5% CO_2_, the extracts, extract-free alginate film formulation, and extract-loaded alginate film formulations were applied at final concentrations of 25–50 μg/mL in ultrapure water and incubated for 24 h. Subsequently, the culture medium was replaced with fresh medium, and 10 μL of MTT solution was added to each well. After 4 h of incubation, the formazan crystals were dissolved using solubilization buffer, and absorbance was measured at 550 and 690 nm (Biotek Epoch, Winooski, VT, USA). Cell viability was expressed as a percentage relative to the untreated control. All experiments were performed in quadruplicate (*n* = 4) [30].

### 2.8. Phytochemical Profiling of the Extracts Using HPLC-DAD

The phenolic constituents of the plant extracts were quantified by HPLC-DAD using a previously reported chromatographic method [35]. Briefly, chromatographic separation was performed on a Nova-Pak C18 reversed-phase column (3.9 ×150 mm, 5 μm) coupled to an Agilent 1260 Infinity HPLC system (Agilent Technologies, Santa Clara, CA, USA) equipped with a diode-array detector. The identification and quantification of the target phenolic compounds were carried out according to their chromatographic and UV-DAD characteristics, using the corresponding reference standards. Quantification was based on calibration curves generated from the relationship between analyte concentration and peak area [35].

### 2.9. Production of the Alginate Film Formulations

The solvent-casting method [59], as a greener and more sustainable option for producing alginate film formulations and an ambient-pressure drying technique, was employed, with the specific method being modified in our laboratory [30]. The extract (300 mg) and sodium alginate (300 mg) were weighed separately and dissolved in deionized water (10 mL) using a magnetic stirrer. The two aqueous solutions were then combined under continuous stirring, and glycerol (1 mL) was added to the mixture as a plasticizer to enhance film flexibility. Petri dishes (9 cm diameter) were used to mold the resulting mixture, into which the mixture was poured. The resulting films were dried at 40 °C in an oven at ambient pressure. Extract free alginate film formulation was also produced using the same procedure.

### 2.10. Characterization of the Film Formulations

To determine the thickness, surface, and cross-sectional morphology of the alginate film formulation, samples were collected from three different parts of each gel and examined using a light microscope (Olympus BX50F-3, Kyoto, Japan). The arithmetic mean of the measurements obtained was calculated. FTIR (Fourier-transform infrared) analysis was applied three times using an IRSpirit spectrometer (Shimadzu Corp, Kyoto, Japan), and the spectra of the film formulations were recorded between 4000–500 cm^−1^ with an average resolution of 4 cm^−1^. In vitro release of alginate film formulations was studied using a spectrophotometric method with a UV-Visible spectrophotometer (Shimadzu 2100S, Kyoto, Japan). Standard-grade regenerated cellulose dialysis bags (6–8 kD) were used in PBS (phosphate-buffered saline, pH 7.4) medium in order to test the release ability of the alginate film formulations. Spectrophotometric measurements were made by measuring the maximum value of the spectrum at a wavelength of 322 nm using extract solutions. Before the in vitro release experiments, measurements were taken at the same wavelength using extract solutions of different concentrations and calibration curves were prepared. Since the release study was designed to quantify the total amount of plant extract, a separate calibration curve was prepared for each extract. Standard solutions in the concentration range of 100–2000 mg/L were prepared for both extracts and analyzed spectrophotometrically at a wavelength of 322 nm. The calibration curve for *T. kotschyanus* was defined as follows:

y = 0.0005x (R^2^ = 0.9998).

For *T. brachicylus*, the calibration curve is as follows:

y = 0.0003x (R^2^ = 0.9998).

The quantity of each extract was determined using the calibration equation specific to that extract. Based on UV–Vis spectral properties and the reproducible absorbance response obtained at this wavelength, a wavelength of 322 nm was used for both extracts. Thus, 322 nm was consistently selected for both calibration standards and release samples. Potential interference effects arising from the alginate film matrix and other formulation components were also evaluated using blank alginate films that did not contain plant extract but were subjected to the same release procedure. The absorbance contribution of the blank formulation at 322 nm was not found compared to the samples containing the extract, which shows that the measured absorbance arises from the UV-absorbing components of the relevant plant extracts. Then, concentrations of extracts in the medium were calculated using prepared calibration curves. 25 mg of each film in 50 mL of PBS medium was used for the release experiments. An in vitro release study was conducted in a thermostatic shaking water bath at 37 °C with a shaking speed of 100 rpm. A known amount of extract-loaded alginate film was placed inside a dialysis bag together with 5 mL of release medium. The dialysis bag was immersed in 45 mL of fresh release medium, which served as the external acceptor compartment. At predetermined time intervals, a 1 mL sample was withdrawn from the external acceptor medium and analyzed using a UV–Vis spectrophotometer. The sampled medium was not replaced with fresh release medium; following the measurement, the same aliquot was returned to the original acceptor compartment. Thus, the total volume of the release medium remained constant throughout the experiment, eliminating the need for any corrections regarding volume reduction or extract loss due to sampling. Samples (1 mL) were taken at predetermined time intervals (at 1 h, 2 h, 3 h, 4 h, 5 h, 6 h and 24 h), and the concentration of the samples was calculated by spectrophotometric method using the calibration curves prepared initially. The percentage of cumulative extract released at each sampling time was calculated by dividing the cumulative amount of extract released at time (t) by the initial amount of extract incorporated into the alginate film and multiplying the result by 100. In vitro release experiments were repeated three times and average values were used for the calculations [60,61].

### 2.11. Statistical Analyses

The survival of *C. elegans* was monitored daily, and the number of surviving nematodes was recorded for each experimental group throughout the observation period. Survival data were analyzed using the Kaplan–Meier method, and Kaplan–Meier survival curves were generated for each group. Survival distributions between groups were compared using the Mantel–Cox log-rank test. Comparisons were performed between the infected control group and the corresponding treatment groups to determine whether treatment affected the survival of *C. elegans*. A *p*-value of < 0.05 was considered statistically significant. Statistical analyses were performed using GraphPad Prism 5.0 software.

## 3. Results

### 3.1. Plants and Extracts

**Figure 1:**
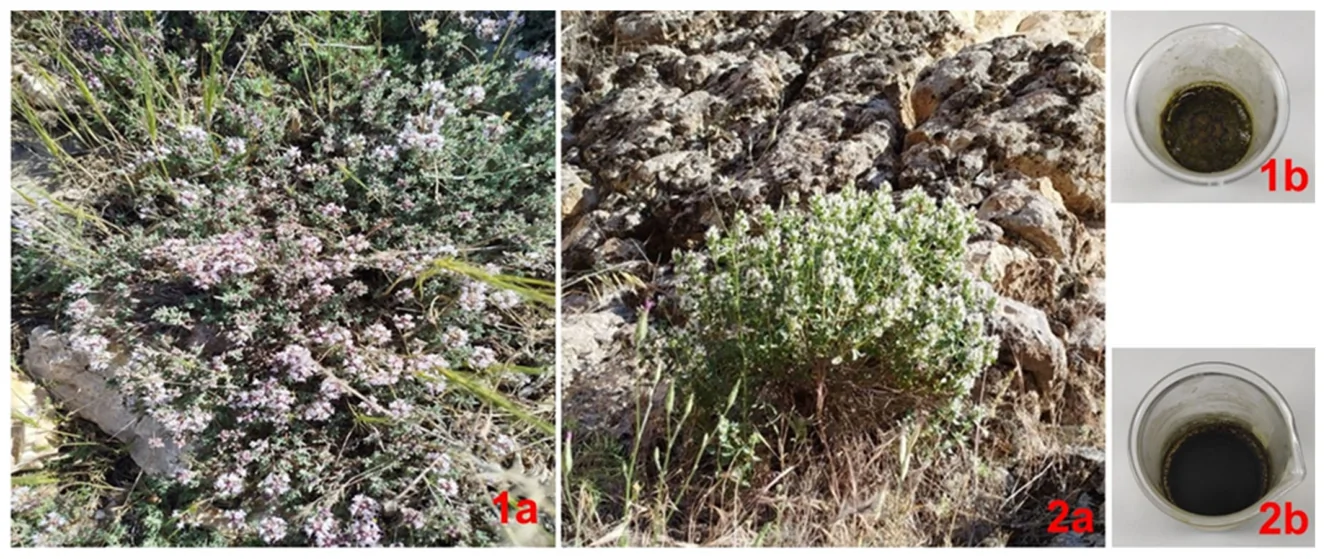
*T. brachychilus* (**1a**), *T. kotschyanus* var. *kotschyanus* (**2a**) (photographs by Ömer Kılıç), and their extracts (**1b**,**2b**).

The plants *T. brachychilus* and *T. kotschyanus* var. *kotschyanus*, along with the ethanol extracts obtained from these plants, are shown in Figure 1.

### 3.2. Antimicrobial Activity

**Figure 2:**
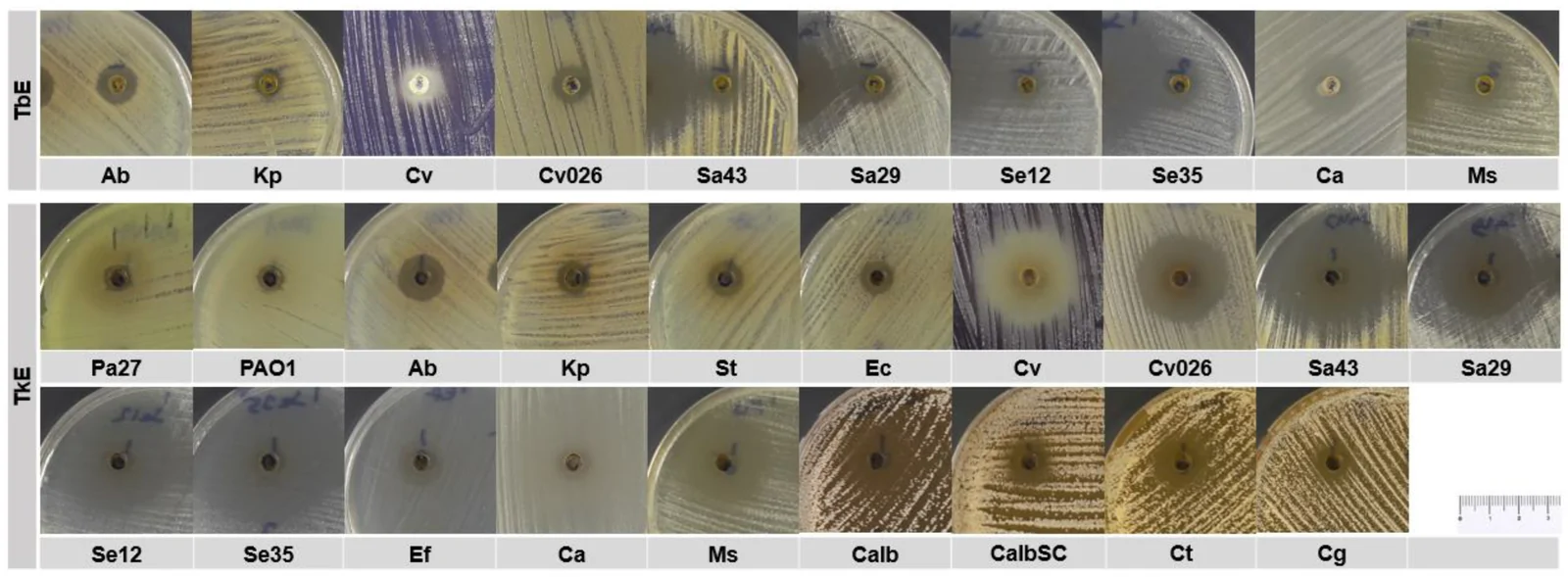
Antimicrobial activity of the extracts determined by the agar well diffusion method. (TbE: *T. brachychilus* extract, TkE: *T. kotschyanus* var. *kotschyanus* extract, Ab: *A. baumannii* ATCC 19606, Kp: *K. pneumoniae* ATCC 4352, Cv: *C. violaceum* ATCC 12472, Cv026: *C. violaceum* CV026, Sa43: *S. aureus* ATCC 43300, Sa29: *S. aureus* ATCC 29213, Se12: *S. epidermidis* ATCC 12228, Se35: *S. epidermidis* ATCC 35984, Ca: *C. acnes* ATCC 11827, Ms: *M. smegmatis* ATCC 19420, Pa27: *P. aeruginosa* ATCC 27853, PAO1: *P. aeruginosa* PAO1, St: *S. Typhimurium* ATCC 14028, Ec: *E. coli* ATCC 25922, Ef: *E. faecalis* ATCC 29212, Calb: *C. albicans* ATCC 90028, CalbSC: *C. albicans* SC5314, Ct: *C. tropicalis* KUEN 1021, Cg: *C. glabrata* ATCC 90030).

The antimicrobial activities of TkE and TbE were evaluated against a broad panel of microorganisms, including Gram-positive bacteria, Gram-negative bacteria, mycobacteria, and yeasts, using the agar well diffusion and broth microdilution methods. Although the agar well diffusion assay did not demonstrate antimicrobial activity against some of the tested microorganisms, the broth microdilution method revealed that both extracts exhibited antimicrobial activity against all tested microorganisms (Table 1, Figure 2). The alginate film formulation containing both extracts was found to retain its antimicrobial activity, particularly against the major microorganisms responsible for skin infections (Table 2).

### 3.3. Effect of Extracts on Quorum Sensing Activity

**Figure 3:**
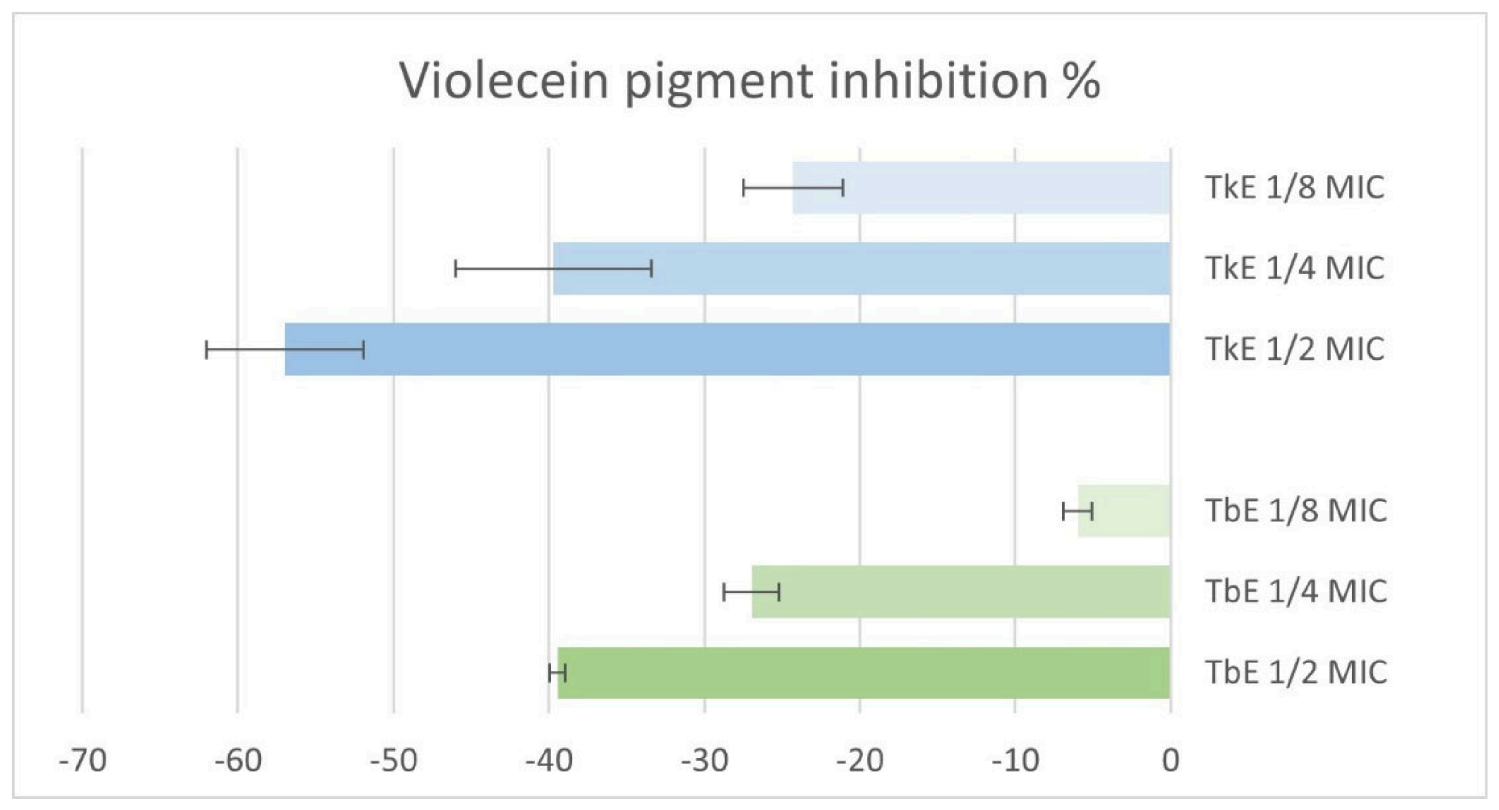
Effects of the extracts on violacein pigment production by *C. violaceum* ATCC 12472 (TbE: *T. brachychilus* extract, TkE: *T. kotschyanus* var. *kotschyanus* extract).

**Figure 4:**
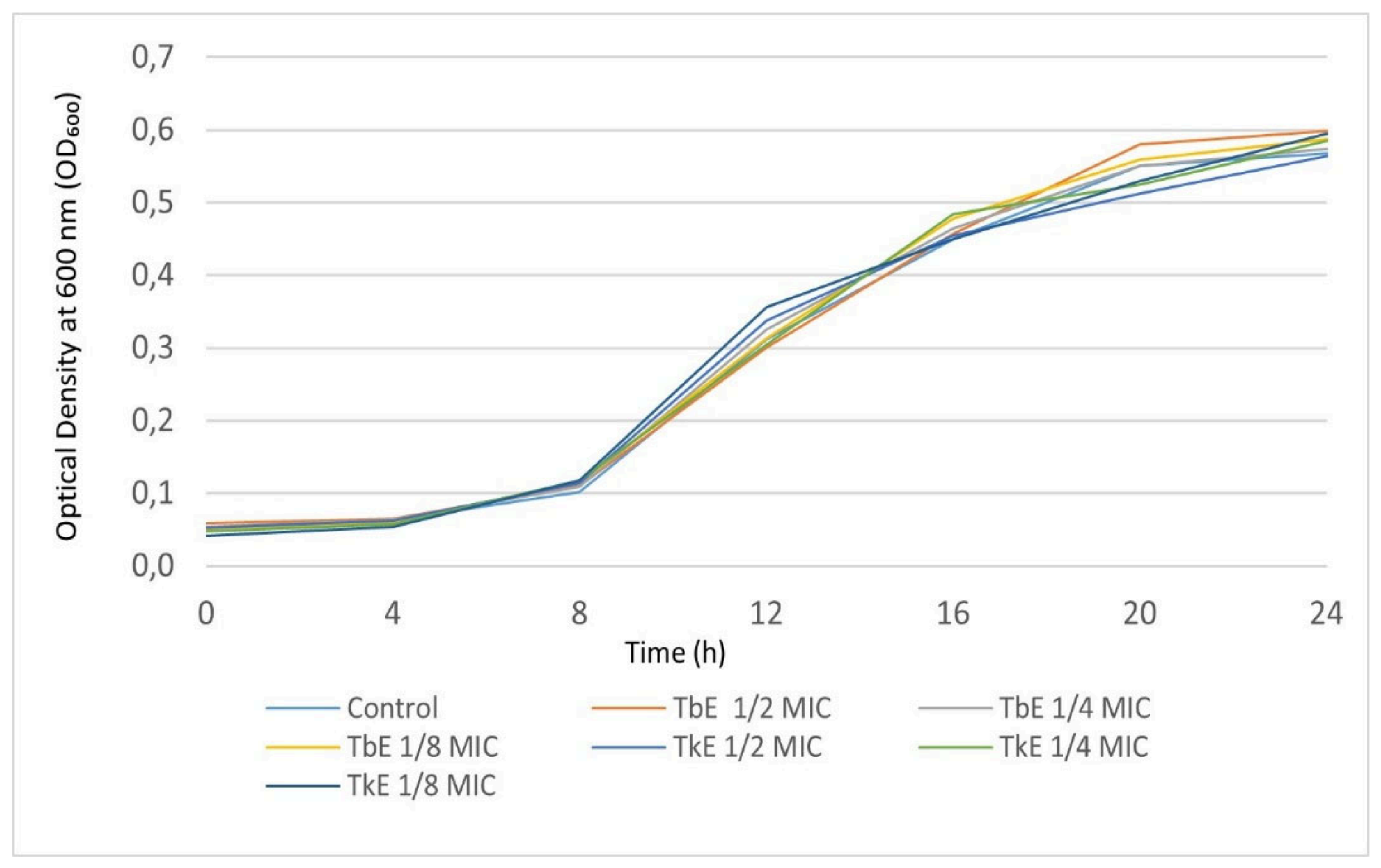
Effect of the extracts at sub-MIC concentrations on the growth of *C. violaceum* ATCC 12472 (TbE: *T. brachychilus* extract, TkE: *T. kotschyanus* var. *kotschyanus* extract).

In the assay investigating violacein pigment production in liquid medium using *C. violaceum* ATCC 12472, the TbE and TkE were found to reduce violacein pigment production in a concentration-dependent manner (Figure 3). At the 1/2 MIC concentration, violacein pigment production was reduced by 39.44% and 56.97% in the presence of TbE and TkE, respectively, while the inhibition rate decreased with decreasing extract concentrations. The growth of the microorganism at the extract concentrations used in the experiments was comparable to that observed under extract-free control conditions (Figure 4).

In the assay performed using *C. violaceum* CV026 on solid medium supplemented with C6-HSL, no colorless zones were observed around the samples.

### 3.4. Effect of Extracts and Alginate Film Formulations on Biofilm Formation

**Figure 5:**
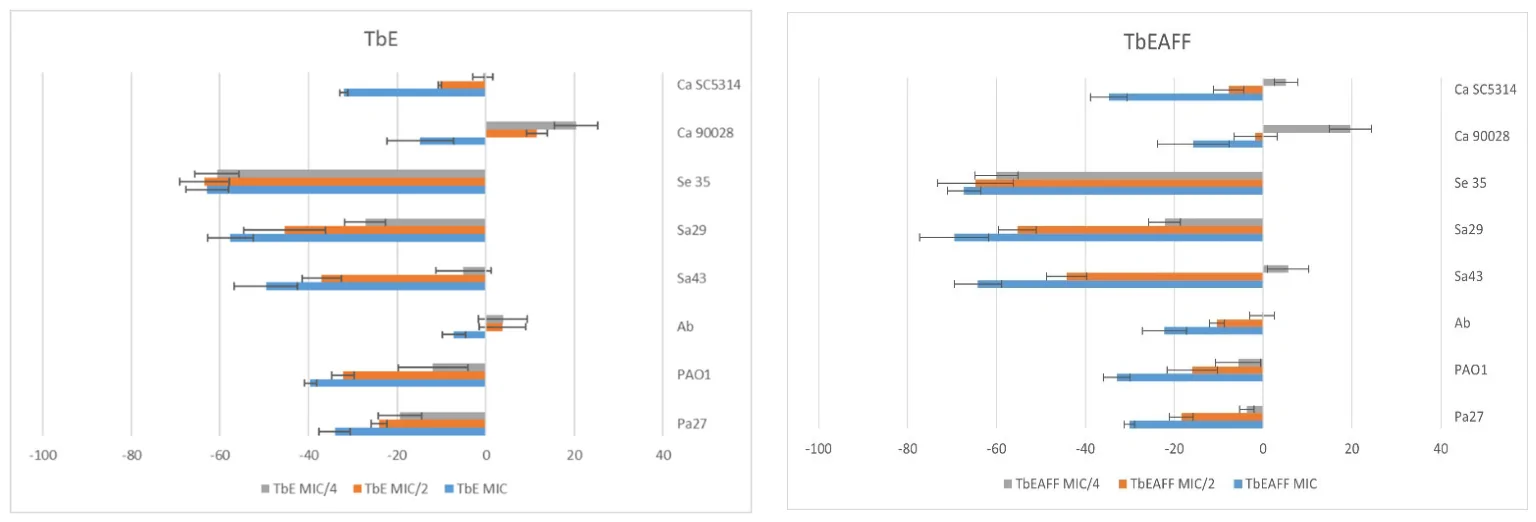

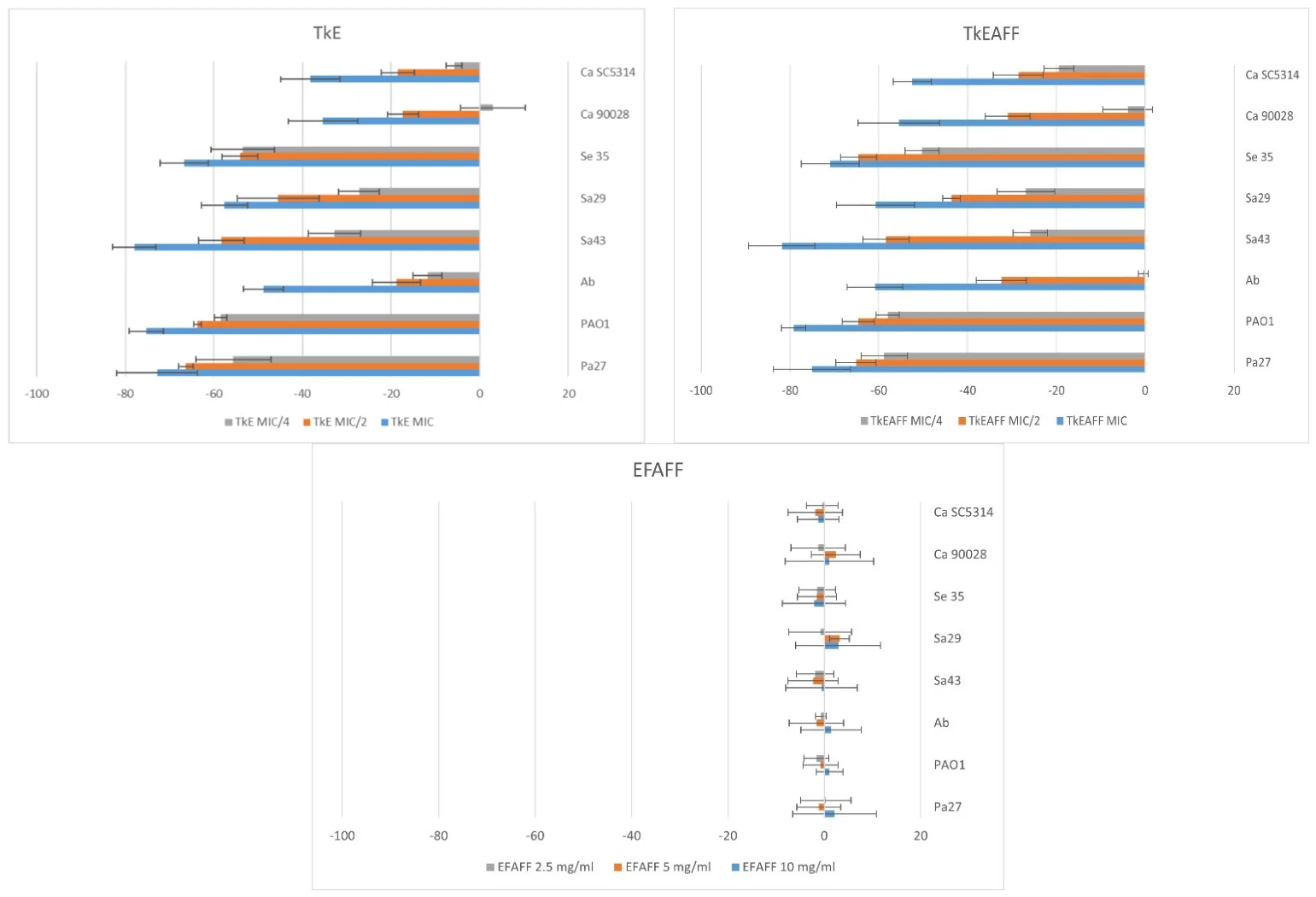
Effect of extracts and alginate film formulations on biofilm formation of microorganisms (negative values shown in the graph represent the percentage decrease in biofilm formation relative to the control. TbE: *T. brachychilus* extract, TbEAFF: *T. brachychilus* extract-loaded alginate film formulation, TkE: *T. kotschyanus* var. *kotschyanus* extract, TkEAFF: *T. kotschyanus* var. *kotschyanus* extract-loaded alginate film formulations, EFAFF: Extract free alginate film formulation, CaSC5314: *C. albicans* SC5314; Ca90028: *C. albicans* ATCC 90028; Se35: *S. epidermidis* ATCC 35984; Sa29: *S. aureus* ATCC 29213; Sa43: *S. aureus* ATCC 43300; Ab: *A. baumannii* ATCC 19606; PAO1: *P. aeruginosa* PAO1; Pa27: *P. aeruginosa* ATCC 27853).

The antibiofilm activities of TbE and TkE were evaluated against different microorganisms with a strong biofilm-forming capacity. Both extracts were found to reduce biofilm formation to varying degrees in a concentration-dependent manner. Furthermore, the alginate film formulations prepared from the extracts exhibited antibiofilm activity similar to that of the extracts (Figure 5). In addition, the extract-free alginate film formulation was found to have a limited effect on biofilm formation.

### 3.5. Detection of Anti-İnfective Effect of Extracts in C. elegans Model

**Figure 6:**
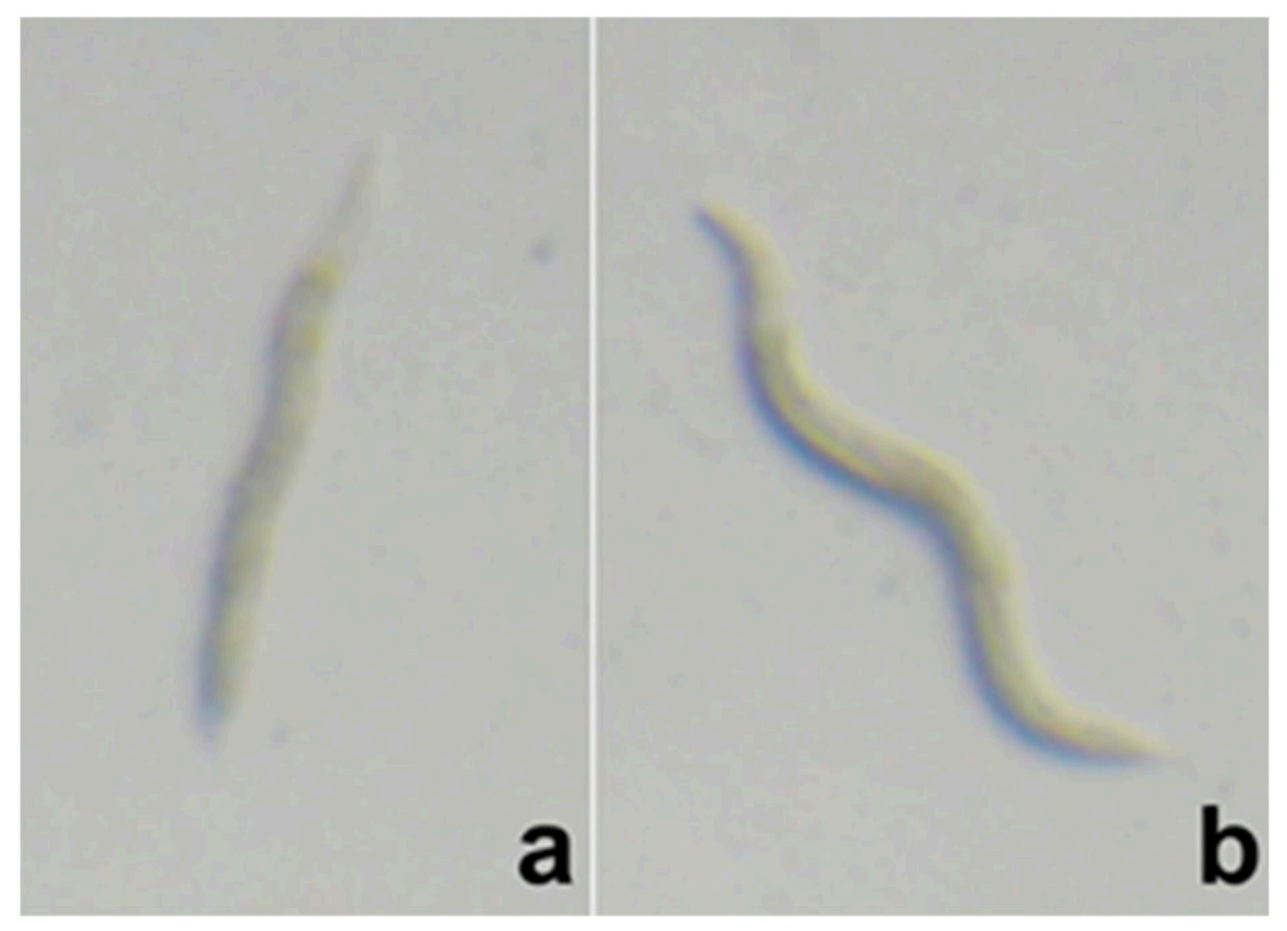
Stereomicroscopic images of dead (**a**) and live (**b**) *C. elegans.*

**Figure 7:**
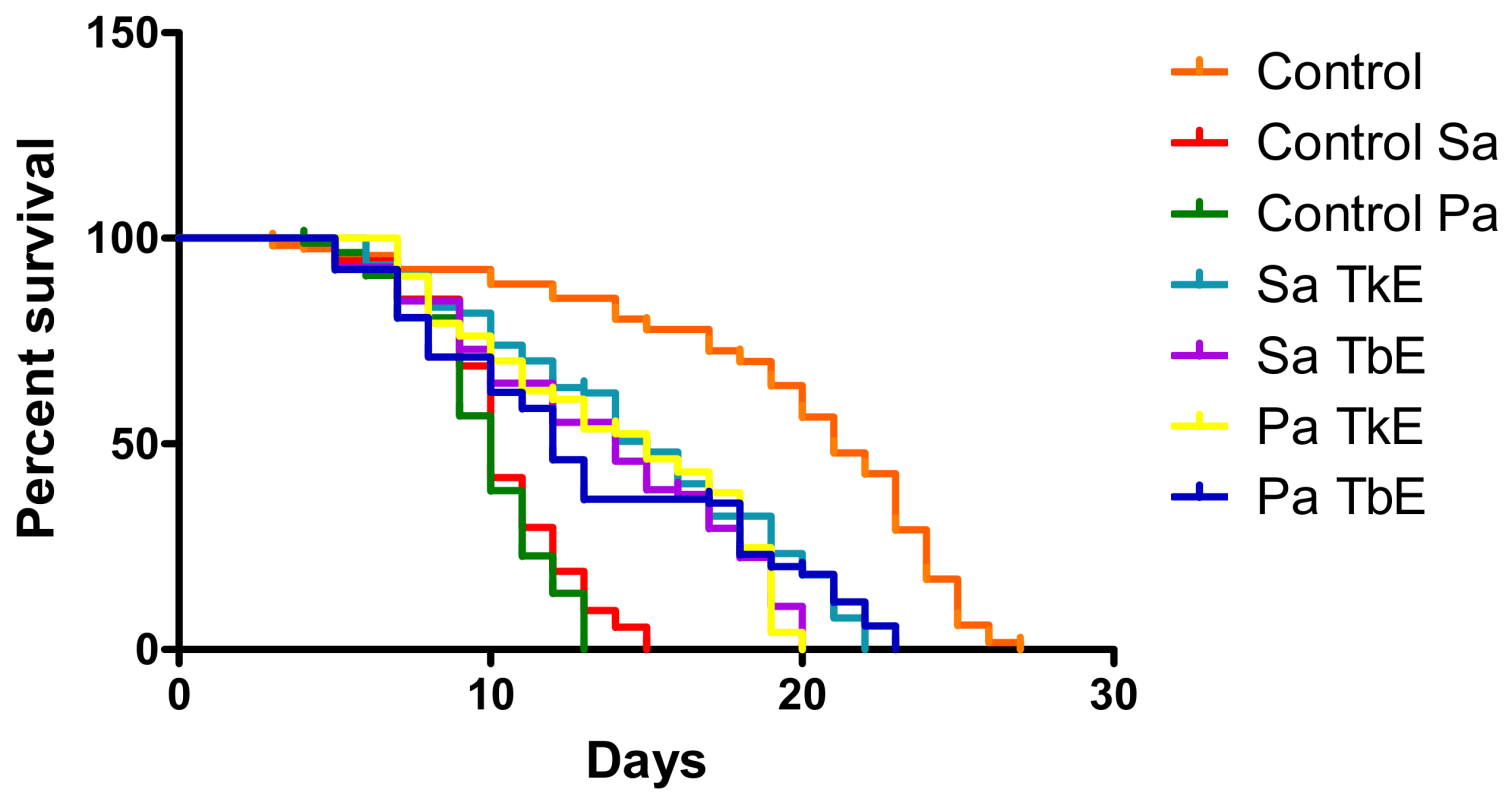
Effects of TbE and TkE on the survival of *C. elegans* infected with *S. aureus* and *P. aeruginosa*. Control: uninfected and untreated group; Control Sa: *S. aureus*-infected group; Control Pa: *P. aeruginosa*-infected group; Sa TkE: *S. aureus*-infected group treated with TbE; Sa TbE: *S. aureus*-infected group treated with TbE; Pa TkE: *P. aeruginosa*-infected group treated with TkE; Pa TbE: *P. aeruginosa*-infected group treated with TbE.

The anti-infective activity of the extracts was investigated against *S. aureus* and *P. aeruginosa* using the *C. elegans* (Figure 6) infection model. Both extracts significantly prolonged the survival of *C. elegans* infected with either pathogen compared with the control groups, demonstrating significant anti-infective activity (*p* < 0.001) (Table 3, Figure 6 and Figure 7).

### 3.6. Cytotoxic Activity of Extracts and Alginate Film Formulations

**Figure 8:**
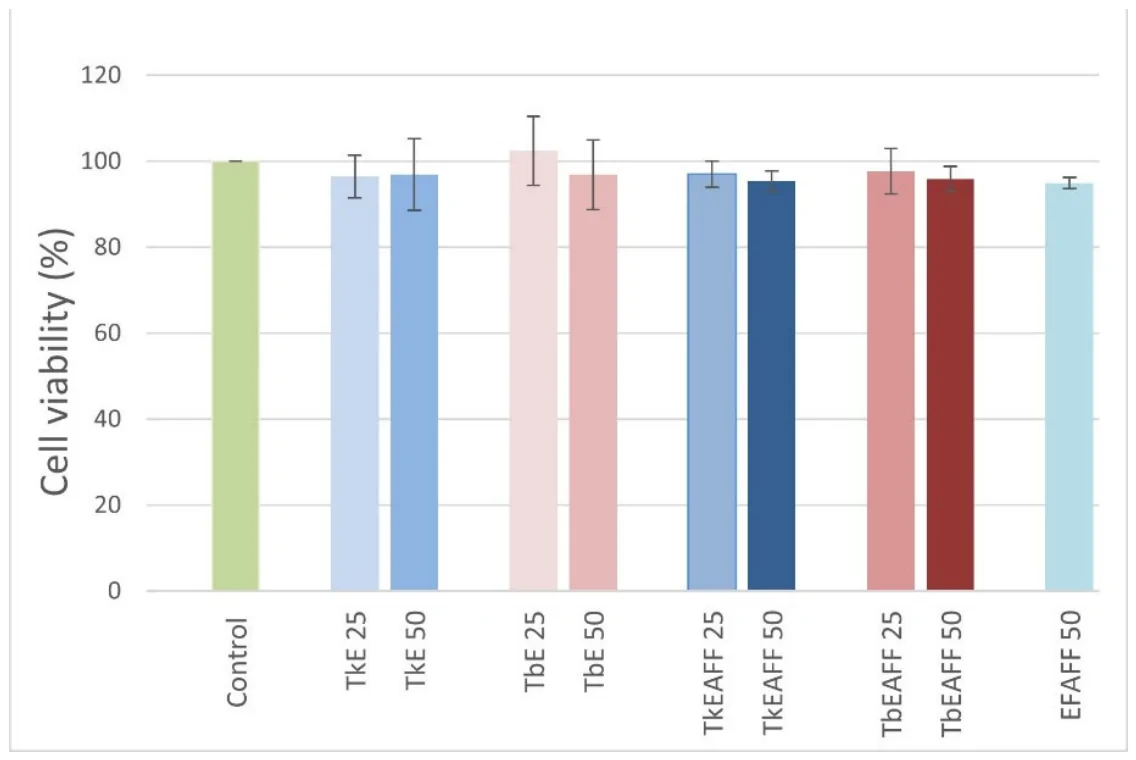
Cytotoxic effect of extracts and alginate film formulations against L929 cells. (TkE: *T. kotschyanus* extract (µg/mL), TbE: *T. brachychilus* extract (µg/mL), TkEAFF: *T. kotschyanus* var. *kotschyanus* extract-loaded alginate film formulation (µg/mL), TbEAFF: *T. brachychilus* extract-loaded alginate film formulation (µg/mL), EFAFF: extract-free alginate film formulation (µg/mL)).

No cytotoxic effects were observed for either the extracts or the extract-loaded alginate film formulations (Figure 8).

### 3.7. Phytochemical Profiling of the Extracts

**Figure 9:**
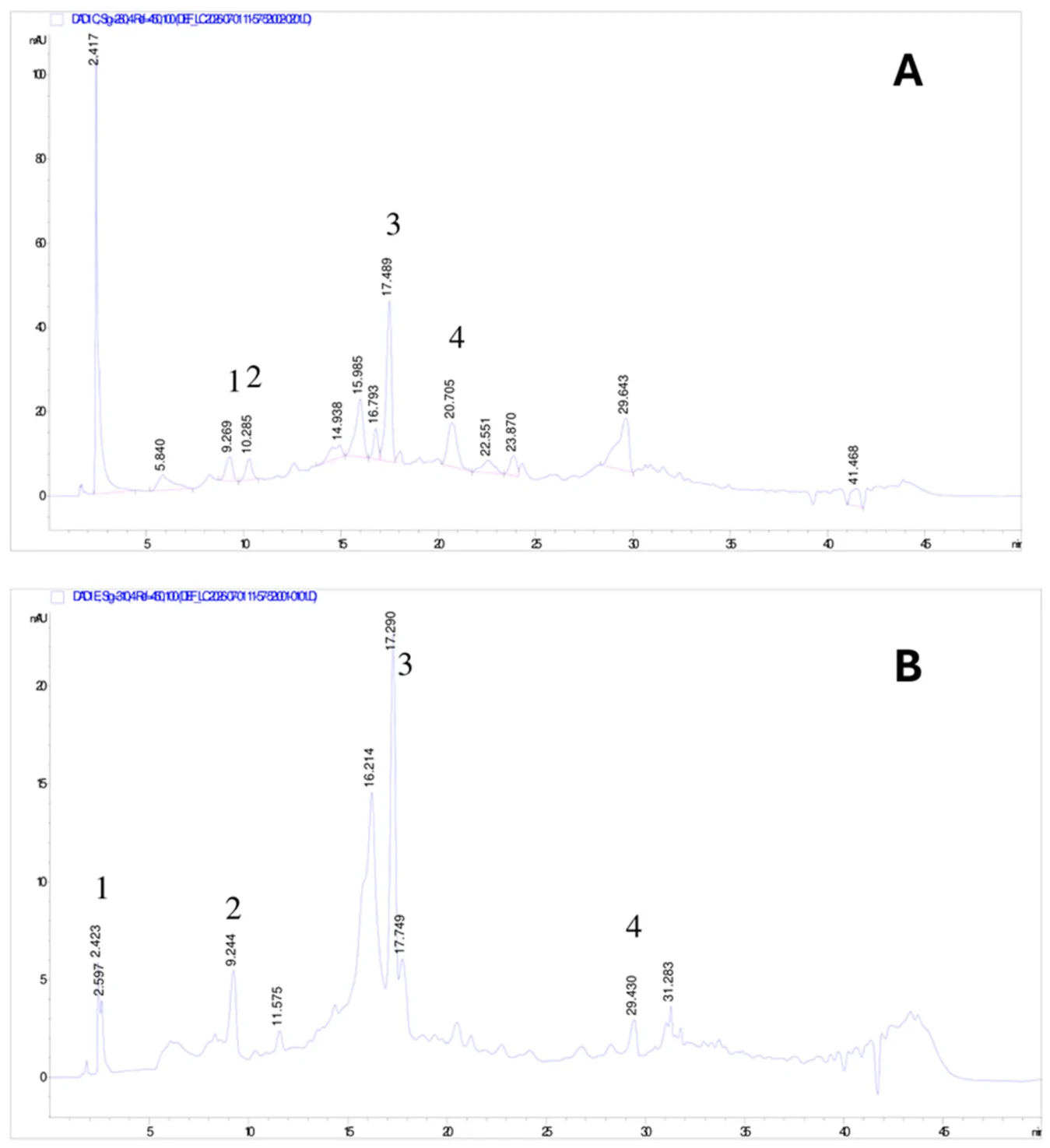
HPLC-DAD chromatogram of ethanol extract of plants (**A**): TbE; 1: caffeic acid; 2: syringic acid; 3: rosmarinic acid; 4: hesperidin; (**B**): TbE; 1: quinic acid; 2: chlorogenic acid; 3: rosmarinic acid; 4: 6-hydroxyflavone).

The phenolic constituents of the plant extracts were quantitatively evaluated by HPLC-DAD. Quantification was performed using calibration curves generated from the relationship between chromatographic peak areas and the corresponding analyte concentrations. In the TkE, caffeic acid, syringic acid, rosmarinic acid and hesperidin were detected at concentrations of 0.803 ± 0.013 μg analyte/mg extract, 1.951 ± 0.333 μg analyte/mg extract, 28.453 ± 0.667 μg analyte/mg extract and 2.265 ± 0.268 μg analyte/mg extract, respectively. The TbE has been found to contain the highest amount of rosmarinic acid. The TbE also contained the four investigated phenolic compounds, with rosmarinic acid being the predominant constituent (19.186 ± 0.224 μg analyte/mg extract), followed by chlorogenic acid (3.869 ± 0.054 μg analyte/mg extract), quinic acid (1.717 ± 0.022 μg analyte/mg extract) and 6-Hydroxyflavone (0.366 ± 0.022 μg analyte/mg extract). Among the quantified compounds, rosmarinic acid was present at the highest concentration in this plant (Figure 9).

### 3.8. Characterization of the Film Formulations

**Figure 10:**
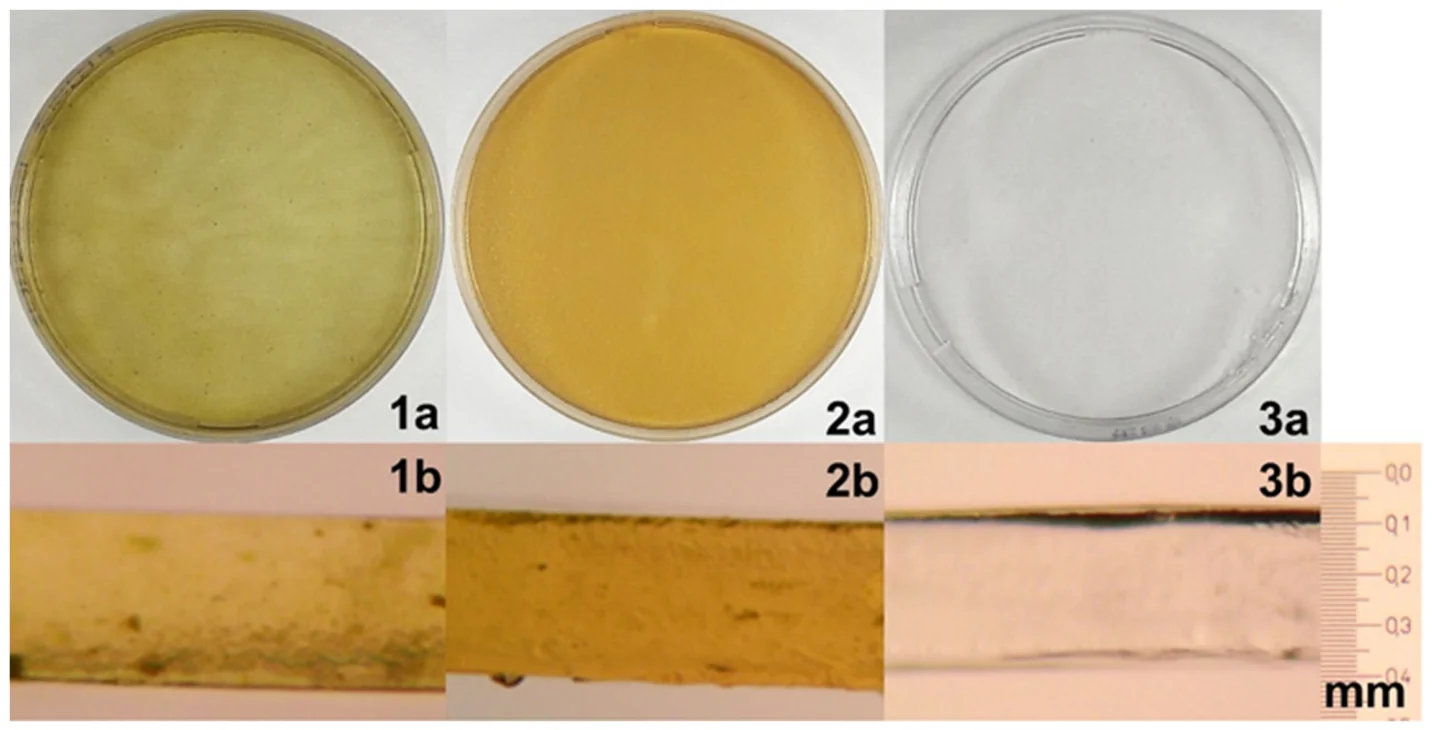
Representative images of alginate film formulations loaded with TbE (**1a**) and TkE (**2a**), the extract-free alginate film formulation (**3a**), and their corresponding cross-sectional images at 40× magnification (**1b**–**3b**).

The macroscopic appearance and cross-sectional morphology of the alginate film formulations with and without TbE and TkE are presented in Figure 10. The thickness of the extract-free alginate film formulation was approximately 0.30 mm, whereas the extract-loaded alginate film formulations had an average thickness of approximately 0.34 mm (Figure 10).

**Figure 11:**
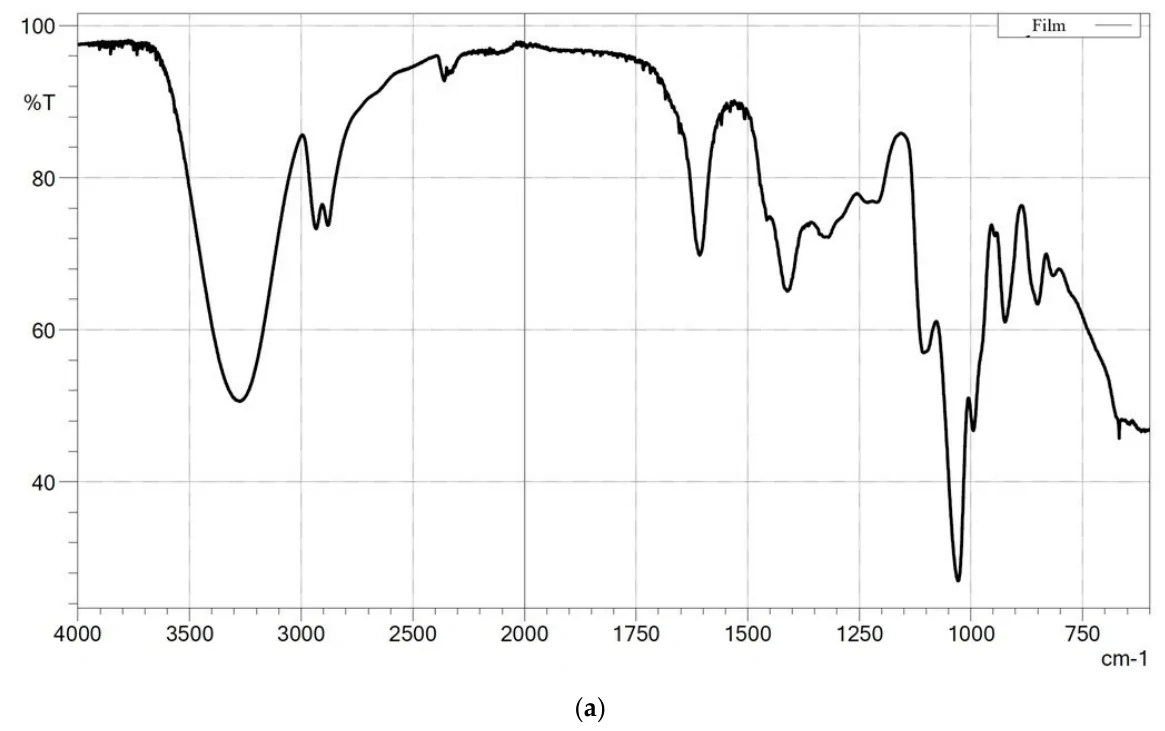

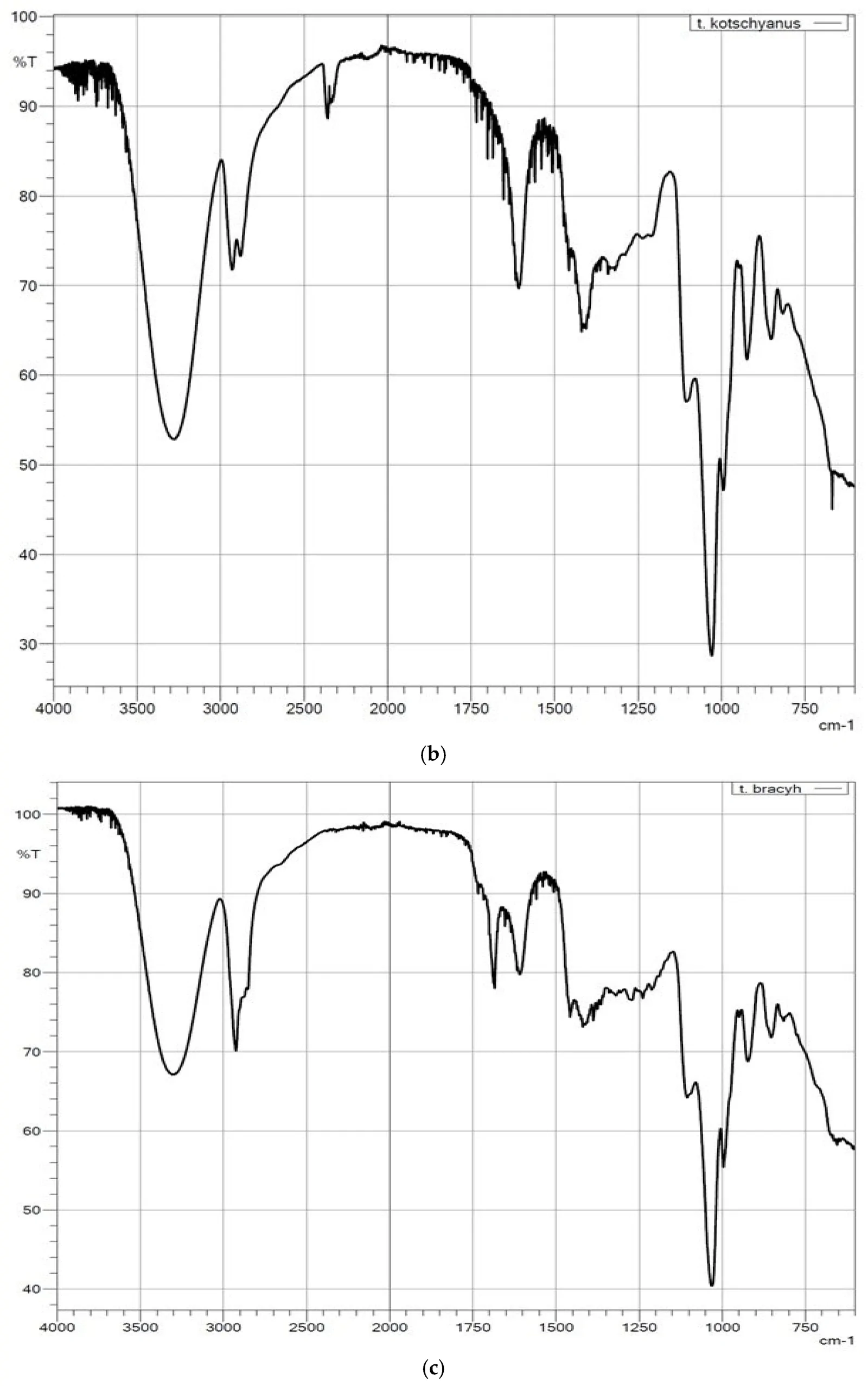
FTIR spectra of extract-free alginate film formulation (**a**) (film), TkE-loaded alginate film formulation (**b**) (t.kotschyanus) and TbE-loaded alginate film formulation (**c**) (t. brach).

The FTIR spectra (Figure 11) were recorded in order to characterize the chemical functionalities of the alginate film structures and show the possible interactions between the bioactive phytochemicals in the plant extracts and the alginate film formulations.

**Figure 12:**
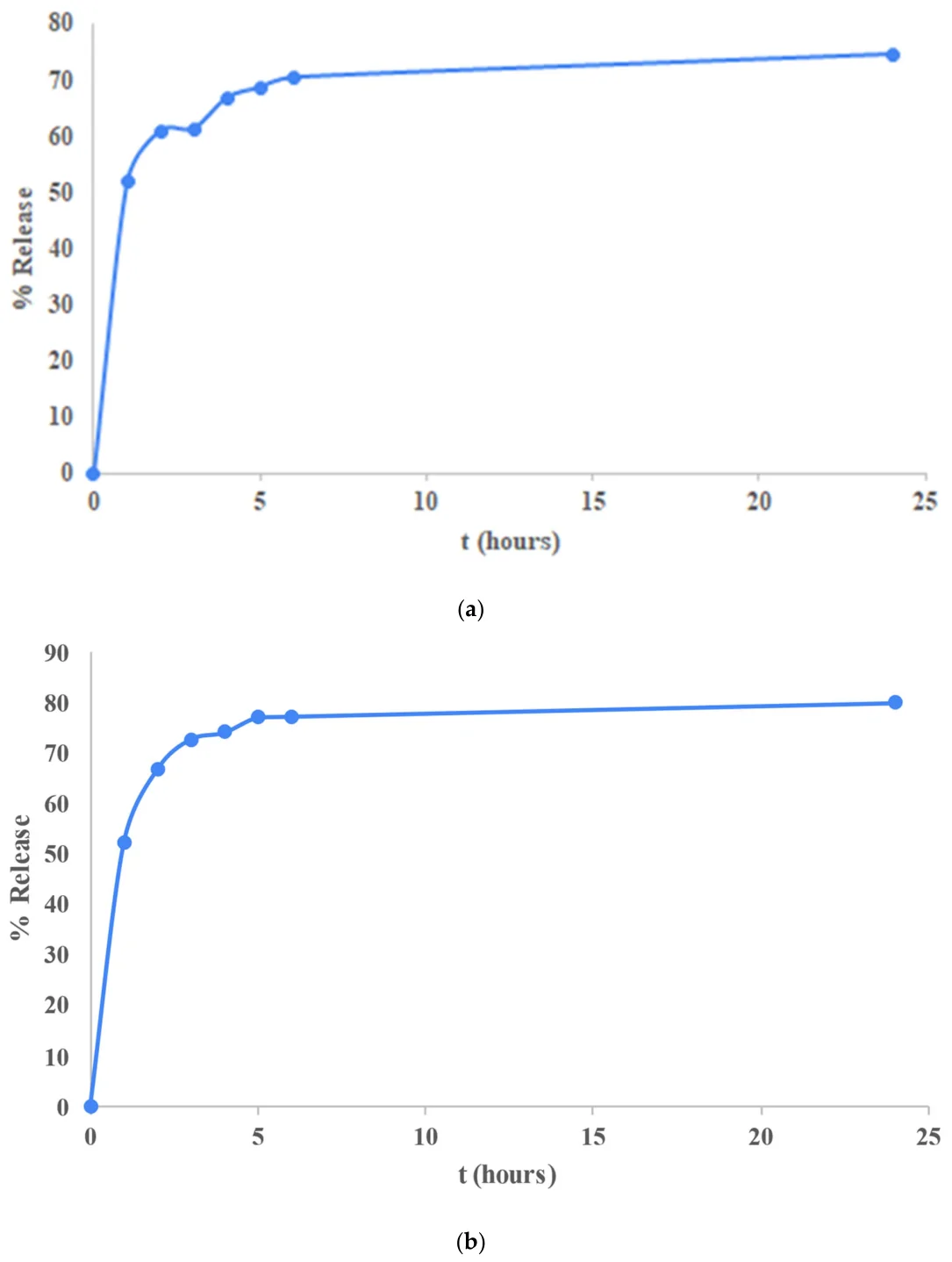
In vitro release profile of alginate film formulations (**a**) TkE-loaded alginate film formulation, (**b**) TbE-loaded alginate film formulation).

In vitro release experiments were conducted to test the release capability of the extracts from the produced formulation. Prior to in vitro release experiments, measurements were performed at a maximum wavelength of 322 nm using extract solutions of various concentrations, and calibration curves were prepared. Spectrophotometric measurements were conducted by determining the maximum spectral value at the same wavelength using the extract solutions. Extract concentrations in the medium were calculated using the prepared calibration curves (Figure 12a,b).

## 4. Discussion

Plants have been extensively investigated in recent years as a rich source of bioactive compounds for addressing various health-related problems. Owing to their diverse secondary metabolites, including flavonoids, alkaloids, and phenolic compounds, plants exhibit remarkable biological activities, particularly in wound healing and the prevention and treatment of microbial infections [62,63]. These secondary metabolites promote tissue repair by modulating multiple biological pathways involved in the different phases of wound healing [64]. Furthermore, these bioactive constituents can be incorporated into a variety of pharmaceutical formulations, enabling the development of effective therapeutic products [65]. Such formulations offer promising alternatives to conventional chemical therapies due to their enhanced stability, biocompatibility, and therapeutic potential [66,67]. Plant-derived metabolites exhibit a wide range of biological activities against microorganisms, including antimicrobial, antibiofilm, anti-quorum sensing, and anti-infective effects. Incorporating these bioactive phytochemicals into appropriate pharmaceutical formulations may facilitate the development of promising therapeutic products, particularly for the prevention and management of wound infections. Such formulations have the potential to enhance treatment efficacy by targeting multiple aspects of microbial pathogenicity while simultaneously supporting the wound-healing process [30,68,69].

In the present study, the antimicrobial activities of extracts from both plant species were evaluated against a broad range of microorganisms using two different methods, including nine Gram-negative bacteria, seven Gram-positive bacteria, one mycobacterial strain, and four yeast strains. This comprehensive approach provided detailed information on the antimicrobial potential of the plant extracts against a diverse range of microorganisms.

Species belonging to the genus *Thymus* have been reported by several researchers to possess strong antimicrobial activity, as well as antibiofilm and anti-quorum sensing (anti-QS) activities. Most of these studies have focused on the essential oils of *Thymus* species [70,71,72,73,74,75]. With regard to the *Thymus* species investigated in the present study, limited information is available on the microbiological and other biological activities of the endemic species *T. brachychilus*. In addition, previous studies on *T. kotschyanus* have predominantly focused on its essential oil rather than its extracts. The essential oil of *T. kotschyanus* has been reported to exhibit antimicrobial activity against various human pathogens, including *E. coli* ATCC 25922, *S. aureus* ATCC 25923, *Bacillus subtilis* ATCC 9372, *K. pneumoniae* ATCC 13183, *P. aeruginosa* ATCC 27853, and *A. baumannii* as well as clinical isolates [76,77]; *Bacillus cereus* ATCC 11778, *Listeria monocytogenes* PTCC 1163, *Salmonella Typhimurium* ATCC 1730, *E. coli* O157:H7 ATCC 25222 [78]; *S. aureus* ATCC 33591 and clinical MRSA isolates [79], *E. coli* ATCC 35218, *S. Typhimurium* ATCC 14028, *S. aureus* ATCC 29213, *B. cereus* ATCC 14579 [80], as well as against several plant pathogens [73].

In previous studies investigating *T. kotschyanus* extracts, the methanolic extract was reported to exhibit antimicrobial activity against *S. aureus* ATCC 25923 and *S. epidermidis* ATCC 12228, whereas no antimicrobial activity was observed against *E. coli* ATCC 25922, *P. aeruginosa* ATCC 27853, *P. mirabilis* ATCC 14153, *K. pneumoniae* ATCC 4352 and *C. albicans* ATCC 10231 [35]. In another study, the methanolic extract exhibited antimicrobial activity against 38 of 52 plant pathogens tested [81]. The chloroform/acetone extract was also reported to be antimicrobial against *E. coli* PTCC 1330, *P. aeruginosa* PTCC 1074, *S. aureus* ATCC 35923, and *B. subtilis* PTCC 1023 [82], while the aqueous extract exhibited antimicrobial activity against *E. coli* PTCC 1763, *P. aeruginosa* PTCC 1047, *K. pneumoniae* PTCC 1053, *S. Typhimurium* PTCC 1609, *P. mirabilis* PTCC 1776, *Enterobacter cloacae* PTCC 1003, and *Shigella dysenteriae* PTCC 1188. In addition, a study investigating the TkE ethanol extract of *T. kotschyanus* used in the present study reported antifungal activity against *C. albicans* ATCC 10231 and 20 clinical *C. albicans* isolates, with MIC and MFC values of 75 and 125 mg/mL, respectively. The ethanol extract was also reported to reduce the expression levels of the CDR1 and CDR2 genes, which are associated with drug resistance [83].

As discussed above, both the essential oil and extracts of *T. kotschyanus* have been reported to exhibit strong antimicrobial activity in previous studies. Although relatively few studies have investigated its ethanol extract, the present study also demonstrated strong antimicrobial activity, consistent with previous findings on this species. In the agar well diffusion assay, the TkE exhibited antimicrobial activity against all tested microorganisms except *P. mirabilis* ATCC 14153 and *S. mutans* ATCC 25175. The TbE, on the other hand, showed antimicrobial activity against *A. baumannii* ATCC 19606, *K. pneumoniae* ATCC 4352, *C. violaceum* ATCC 12472, *C. violaceum* CV026, *S. aureus* ATCC 43300, *S. aureus* ATCC 29213, *S. epidermidis* ATCC 12228, *S. epidermidis* ATCC 35984, *C. acnes* ATCC 11827, and *M. smegmatis* ATCC 19420 (Table 1, Figure 2). In the broth microdilution assay, both extracts exhibited antimicrobial activity against all tested microorganisms, although the magnitude of activity varied among the microorganisms. The MIC values ranged from 0.029 to 7.5 mg/mL for the TbE and from 0.029 to 1.88 mg/mL for the TkE. The MBC (for bacteria)—MFC (for yeasts) values ranged from 0.23–>15 mg/mL for the TbE and from 0.06 to 3.75 mg/mL for the TkE (Table 1). Differences in antimicrobial activity between agar well diffusion and broth microdilution assays have also been reported in some cases [30]. These differences may be attributed to variations in the composition and characteristics of the culture media used in the two assays, as well as differences in the susceptibility of individual microorganisms to the tested extracts. The antimicrobial activity results demonstrated that both extracts exhibited broad-spectrum antimicrobial activity against several microorganisms commonly associated with skin-related health problems, including *S. aureus*, *S. epidermidis*, *P. aeruginosa*, *A. baumannii*, and *C. acnes*. These findings suggest that both extracts may have potential for use in applications targeting microorganisms associated with skin infections and related health conditions.

Two of the most commonly used methods in the literature for evaluating the effects of extracts on QS activity were employed [49]. In the assay conducted in liquid medium using *C. violaceum* ATCC 12472, the extracts caused a concentration-dependent reduction in violacein pigment production (Figure 3), whereas no activity was detected for the extracts in the assay performed using *C. violaceum* CV026 on solid medium supplemented with C6-HSL. The discrepancy between the two assays may be attributed to differences in the experimental conditions and the mechanisms assessed by each method. In particular, the limited diffusion of some bioactive constituents of the extracts through the solid medium may have contributed to the absence of detectable activity in the CV026 assay. While the assay using *C. violaceum* ATCC 12472 primarily evaluates QS-regulated violacein production, the assay employing *C. violaceum* CV026 assesses whether the violacein response induced by exogenous C6-HSL through CviR is inhibited. Since the extracts used in the *C. violaceum* ATCC 12472 assay did not affect microbial growth compared with the extract-free control (Figure 4) but reduced violacein pigment production, these findings suggest that the extracts may interfere with QS-regulated violacein production.

There are insufficient data regarding the effects of either of these two plant species on biofilm formation by microorganisms. In a study investigating six different *Thymus* essential oils, including three different oils of *Thymus vulgaris* L., two different oils of *Thymus zygis* L., and one oil of *Thymus satureioides* Coss., the essential oils were shown to inhibit biofilm formation by *S. mutans* ATCC 700610, particularly at concentrations of 2 MIC, MIC, and 1/2 MIC [84].

In the present study, microorganisms that are major potential causative agents of skin infections and possess a strong capacity to form biofilms were specifically selected to evaluate the effects of the extracts on biofilm development. To distinguish antibiofilm activity from direct antimicrobial effects, extract concentrations corresponding to the MIC, 1/2 MIC, and 1/4 MIC were selected for each microorganism. The TbE altered biofilm formation by −7.23% to −62.93% at the MIC, +11.56% to −63.51% at 1/2 MIC, and +20.41% to −60.71% at 1/4 MIC concentrations (+ values indicate an increase in biofilm formation, whereas − values indicate a reduction in biofilm formation). The strongest antibiofilm effect of the TbE was observed against *Staphylococcus* species. The TkE altered biofilm formation by −35.37% to −77.89% at the MIC, −17.35% to −66.34% at 1/2 MIC, and +3.06% to −58.49% at 1/4 MIC concentrations (+ values indicate an increase in biofilm formation, whereas − values indicate a reduction in biofilm formation). The TkE exhibited its strongest antibiofilm activity against all tested Gram-positive and Gram-negative bacteria. For both extracts, the effects on biofilm formation varied depending on the extract concentration (Figure 5). Since data on the effects of these plant extracts on biofilm formation are limited, the present study provides valuable information to address this gap in the literature. Notably, the TbE exhibited strong antibiofilm activity, particularly against Gram-positive bacteria, whereas the TkE showed strong antibiofilm activity against both Gram-positive and Gram-negative bacteria.

Anti-infective activity was evaluated using a *C. elegans* infection model. In this model, the effects of the tested extracts on preventing nematode mortality and prolonging lifespan following infection with *S. aureus* and *P. aeruginosa* were investigated. The survival of infected nematodes was compared with that of the corresponding control groups, and the protective effects of extract treatment against infection-associated mortality were assessed. In the anti-infective activity assessment using the *C. elegans* infection model, both extracts significantly prolonged the lifespan of nematodes infected with *S. aureus* and *P. aeruginosa* (*p* < 0.001) (Table 3, Figure 7). These findings suggest that the extracts may exert not only direct antimicrobial activity but also a protective anti-infective effect that may contribute to host survival under infectious conditions.

Due to the activities identified for the extracts, it is important to evaluate the cytotoxicity of both the extracts and the formulation intended for extract loading prior to formulation studies. For this purpose, the cytotoxic activities of the extracts and the extract-free formulation were evaluated in the L-929 (ATCC CCL-1) cell line using the MTT assay. Both extracts, the extract-loaded alginate film formulations, and the extract-free alginate film formulation were found to be non-cytotoxic (Figure 8). The absence of cytotoxic properties in this formulation system has also been demonstrated in previous studies [30].

The compositions of the extracts were analyzed by HPLC-DAD. Based on the quantities detected, the major compounds identified in the TbE were rosmarinic acid, chlorogenic acid, quinic acid, and 6-hydroxyflavone, whereas rosmarinic acid, hesperidin, syringic acid, and caffeic acid were identified as the major compounds in the TkE (Figure 9). Limited information is available on the ethanol extracts of these plant species. The ethanol extract of *T. kotschyanus* Boiss. has been reported to contain various bioactive compounds, including coumarins, flavonoids, alkaloids, tannins, phenolic compounds, carbohydrates, proteins, and saponins [85]. Rosmarinic acid, which was identified as a major compound in both extracts, is well known for its strong antimicrobial properties. Rosmarinic acid has been reported to exhibit antimicrobial activity against methicillin-resistant *S. aureus* (MRSA) and *S. aureus* and to act synergistically with vancomycin, ofloxacin, and amoxicillin against these microorganisms [86,87]. It has also been reported to exhibit antimicrobial activity against various other human pathogens, as well as antibiofilm activity, particularly against certain *Candida* species, and anti-QS activity against *Aeromonas hydrophila* [87,88]. Chlorogenic acid has also been shown to exhibit antimicrobial activity against various microorganisms, including *S. aureus*, *P. aeruginosa*, *E. coli*, *K. pneumoniae*, *A. baumannii*, and *C. albicans*, as well as antibiofilm activity against *S. aureus*, *P. aeruginosa*, and *C. albicans* [89,90,91,92]. Quinic acid has been reported to exhibit antimicrobial activity against *S. aureus* and *E. coli* and antibiofilm activity against *P. aeruginosa* [93,94,95]. 6-Hydroxyflavone has been reported to enhance biofilm formation in *A. baumannii* [96], have no significant effect on biofilm formation in *C. albicans* [97], and inhibit biofilm formation in *E. coli* O157:H7 [98]. Hesperidin has been reported to exhibit antimicrobial, antibiofilm, and anti-quorum sensing (anti-QS) activities against *Aeromonas hydrophila* [99], as well as antimicrobial activity against *Listeria monocytogenes* and *S. aureus* [100,101]. It has also been shown to exhibit antimicrobial and antibiofilm activities against *S. aureus* ATCC 33591 [102] and antimicrobial activity against *P. aeruginosa* and MRSA [103]. Syringic acid has been reported to exhibit antibiofilm and anti-QS activities against *S. epidermidis* ATCC 35984 and clinical *S. epidermidis* isolates [104], as well as antibiofilm activity against *S. marcescens* [105]. Caffeic acid has been reported to exhibit antimicrobial activity against *S. aureus* [106,107], as well as anti-QS and antibiofilm activities against *S. aureus* [108]. In addition, caffeic acid has been reported to possess antiviral activity [109]. Taken together, the major compounds identified in the extracts exhibit a broad range of biological activities, including antimicrobial, antibiofilm, and anti-QS effects. Therefore, the strong microbiological activities observed for our extracts may be, at least in part, attributable to the presence of these bioactive compounds. However, considering the complex composition of plant extracts, synergistic or additive interactions among these constituents may also contribute to the observed biological effects.

In our study, alginate film formulations with potential to support the treatment of skin infections were developed using TbE and TkE, based on their potent microbiological activities and lack of cytotoxic properties. The developed alginate film formulations were characterized using light microscopy, FTIR analysis, and in vitro release studies. Examination of the cross-sections obtained by light microscopy revealed that the extract-free alginate film formulation had a thickness of approximately 0.30 mm, whereas the extract-loaded alginate film formulations had a thickness of approximately 0.34 mm (Figure 10). In the FTIR spectra (Figure 11), there is a wide absorption band between 3000–3500 cm^−1^, which shows the stretching of OH groups of the alginate matrix. C-H bands are seen at around 2900 cm^−1^, while asymmetric and symmetric stretching vibrations of the COO− groups are present at around 1620 cm^−1^ and 1420 cm^−1^. C-O-C stretching is also seen at around 1040 in the spectra as a characteristic of the alginate structure [61,110,111,112,113,114,115]. Figure 11 also shows that there are only small changes in the peak positions between empty and extract-loaded film formulations as a sign of the physical dispersion of the material and physical interaction between the phytochemical content of the extracts and the alginate matrix of the film. Consistent with our previous study, FTIR results indicate that the extract incorporated into the formulation physically adheres to the film matrix primarily through non-covalent interactions such as hydrogen bonding without causing significant changes to the film’s fundamental chemical structure, which is favorable for pharmaceutical formulations [30]. Figure 12 shows that there is a gradual and relatively slow release of the bioactive phytochemicals of the extracts for both of the film formulations. Slow and sustainable drug release of a potential wound dressing is important to improve wound healing through the increase in the duration of the release of the active ingredients. Long release time also improves the compliance of the therapeutic application to the patients since it would increase the application time before replacement [30,113,114]. The antimicrobial and antibiofilm activities of the developed alginate film formulations were evaluated using the same methods employed for the extracts. Microorganisms commonly associated with skin infections were specifically selected for the evaluation of the antimicrobial and antibiofilm activities of the alginate film formulations. The antimicrobial activity was determined using the agar well diffusion method, while the antibiofilm activity was assessed using the same method employed to evaluate the antibiofilm activity of the extracts. The developed alginate film formulations retained both their antimicrobial and antibiofilm activities, and these activities were similar to those observed for the extracts alone (Table 2, Figure 5). This finding is important as it indicates that the developed formulation system did not adversely affect the biological activities of the extracts.

Several limitations of the study should be considered. Since no previous studies have investigated the microbiological activities or formulation of the endemic species *T. brachychilus* and *T. kotschyanus* var. *kotschyanus* in this context, the antimicrobial and antibiofilm activities of the extracts were evaluated against a broad spectrum of microorganisms. In addition, their anti-QS and anti-infective activities were also investigated. Therefore, the extracts and the developed alginate film formulations were not evaluated against clinical isolates in the present study. Furthermore, further studies involving gene expression analyses to elucidate the anti-QS activity of the extracts at the molecular level, as well as evaluation of the developed alginate film formulations in in vivo wound infection models and assessment of their wound-healing activity, would be valuable for providing further evidence of their therapeutic potential.

## 5. Conclusions

In this study, the antimicrobial activity of ethanol extracts obtained from the endemic species *T. brachychilus* and *T. kotschyanus* var. *kotschyanus* were evaluated against a broad microbiological spectrum, and their antibiofilm, anti-QS, anti-infective, and cytotoxic activities were investigated. Both extracts exhibited strong antimicrobial, antibiofilm, and anti-infective activities, showed anti-QS potential, and were found to contain rosmarinic acid as a major compound based on HPLC-DAD analysis. Given their potent microbiological activities and lack of cytotoxicity, alginate film formulations were developed with the potential to support the treatment of skin infections. The alginate film formulations were characterized by light microscopy, FTIR analysis, and in vitro release studies, and their antimicrobial, antibiofilm, and cytotoxic properties were evaluated. The developed formulation was found to be non-cytotoxic, while its antimicrobial and antibiofilm activities were similar to those of the extracts. These findings indicate that the developed formulation system possesses suitable characteristics for preserving and enabling the biological activities of the extracts. Although the in vitro findings of our study indicate that the alginate gel formulations loaded with TbE and TkE have considerable potential to support wound treatment, further studies are needed to demonstrate the efficacy of such herbal formulations in in vivo models, investigate the mechanisms underlying the activities of the constituents present in the extracts, and determine their interactions with antimicrobial drugs used in the treatment of wound infections. These studies will contribute to a better understanding of the therapeutic potential of these formulations.

## Figures and Tables

**Table 1:** Antimicrobial activity of extracts on various microorganisms.

		ZD (mm) ± SD/MIC (mg/mL)/MBC (mg/mL, for Bacteria)—MFC (mg/mL, for Yeasts)	
Microorganisms		TbE	TkE
**Gram-negative bacteria**	*Pseudomonas aeruginosa* ATCC 27853	0/1.88/7.5	9.06 ± 0.10/1.88/3.75
	*Pseudomonas aeruginosa* PAO1	0/1.88/3.75	7.51 ± 0.06/0.94/1.88
	*Acinetobacter baumannii* ATCC 19606	13.19 ± 0.08/0.94/1.88	13.74 ± 0.10/0.47/0.94
	*Proteus mirabilis* ATCC 14153	0/0.94/7.5	0/0.94/1.88
	*Klebsiella pneumoniae* ATCC 4352	7.65 ± 0.17/0.94/7.5	10.41 ± 0.05/0.94/1.88
	*Salmonella Typhimurium* ATCC 14028	0/0.94/1.88	10.45 ± 0.09/0.94/1.88
	*Escherichia coli* ATCC 25922	0/1.88/3.75	10.11 ± 0.15/0.47/0.94
	*Chromobacterium violaceum* ATCC 12472	10.88 ± 0.12/0.12/0.23	27.74 ± 0.08/0.06/0.12
	*Chromobacterium violaceum* CV026	11.61 ± 0.03/0.12/0.47	29.28 ± 0.03/0.029/0.06
**Gram-positive bacteria**	*Staphylococcus aureus* ATCC 43300	13.82 ± 0.04/0.12/3.75	38.06 ± 0.09/0.23/0.47
	*Staphylococcus aureus* ATCC 29213	13.12 ± 0.03/0.23/3.75	27.26 ± 0.04/0.23/0.47
	*Staphylococcus epidermidis* ATCC 12228	11.22 ± 0.04/0.47/0.94	36.47 ± 0.07/0.23/0.47
	*Staphylococcus epidermidis* ATCC 35984	13.29 ± 0.16/0.47/0.94	41.94 ± 0.05/0.23/0.47
	*Enterococcus faecalis* ATCC 29212	0/1.88/>15	15.10 ± 0.12/0.47/1.88
	*Streptococcus mutans* ATCC 25175	0/0.47/3.75	0/0.47/1.88
	*Cutibacterium acnes* ATCC 11827	19.07 ± 0.05/0.029/0.06	41.10 ± 0.07/0.029/0.06
**My.**	*Mycobacterium smegmatis* ATCC 19420	11.93 ± 0.04/0.12/0.23	24.05 ± 0.10/0.12/0.23
**Yeasts**	*Candida albicans* ATCC 90028	0/0.47/3.75	10.15 ± 0.06/0.47/0.94
	*Candida albicans* SC5314	0/1.88/3.75	12.53 ± 0.16/0.23/0.47
	*Candida tropicalis* KUEN 1021	0/3.13/6.25	13.90 ± 0.06/0.78/1.56
	*Candida glabrata* ATCC 90030	0/0.94/3.75	10.41 ± 0.15/0.47/0.94

ZD: inhibition zone diameter, MIC: minimum inhibitory concentration, MBC: minimum bactericidal concentration, MFC: minimum fungicidal concentration, TbE: *T. brachychilus* extract, TkE: *T. kotschyanus* var. *kotschyanus* extract, My.: Mycobacterium.

**Table 2:** Antimicrobial activity of alginate film formulations using the agar well diffusion method.

	ZD (mm) ± SD	
Microorganisms	TbEAFF	TkEAFF
*P. aeruginosa* ATCC 27853	0	8.72 ± 0.14
*A. baumannii* ATCC 19606	11.28 ± 0.12	11.55 ± 0.18
*E. coli* ATCC 25922	0	8.25 ± 0.08
*S. aureus* ATCC 43300	12.74 ± 0.17	31.35 ± 0.21
*S. aureus* ATCC 29213	8.25 ± 0.10	25.22 ± 0.13
*S. epidermidis* ATCC 12228	9.57 ± 0.17	37.11 ± 0.20
*E. faecalis* ATCC 29212	0	15.75 ± 0.0.9
*C. acnes* ATCC 11827	17.48 ± 0.12	37.41 ± 0.19
*C. albicans* ATCC 90028	0	8.58 ± 0.18

ZD: inhibition zone diameter, TbEAFF: *T. brachychilus* extract-loaded alginate film formulation, TkEAFF: *T. kotschyanus* var. *kotschyanus* extract-loaded alginate film formulation.

**Table 3:** Anti-infective effect of extracts in *C. elegans* model.

Group	*n*	Median Survival (Day)	95% CI	*p*
Control	118	21	-	-
Control Sa	74	10	-	-
Contro Pa	88	10	-	-
Sa TbE	77	14	−0.018–1.45	<0.0001 *
Sa TkE	85	15	−0.06–1.40	<0.0001 *
Pa TbE	97	12	0.08–1.59	<0.0001 **
Pa TkE	104	15	−0.08–1.42	<0.0001 **

* Compared with the “Control Sa” value; ** compared with the “Control Pa” value. Control: uninfected and untreated group; Control Sa: *S. aureus*-infected group; Control Pa: *P. aeruginosa*-infected group; Sa TkE: *S. aureus*-infected group treated with TkE; Sa TbE: *S. aureus*-infected group treated with TbE; Pa TkE: *P. aeruginosa*-infected group treated with TkE; Pa TbE: *P. aeruginosa*-infected group treated with TbE.

## Data Availability

The data obtained from this study are presented in the article. Further detailed data are available from the corresponding author upon reasonable request.

## Abbreviations

The following abbreviations are used in this manuscript:

CLSI	Clinical & Laboratory Standards Institute
FTIR	Fourier-transform infrared
SEM	Scanning electron microscopy
MIC	Minimum inhibitory concentration
